# Visualizing early allograft rejection: an M1 macrophage-specific GLUT1 probe predicts TCMR onset in renal transplantation

**DOI:** 10.3389/fimmu.2025.1670370

**Published:** 2025-10-02

**Authors:** Zhaoxiang Wang, Fanchao Wei, Dayuan Huang, Ruochen Qi, Shichao Han, Changhong Shi, Hongtao Song, Yuxuan Du, Zhengxuan Li, Lang Li, Jingliang Zhang, Shuaijun Ma, Weijun Qin

**Affiliations:** ^1^ Department of Urology, Xijing Hospital, Air Force Medical University, Xi’an, Shaanxi, China; ^2^ Division of Cancer Biology, Laboratory Animal Center, Air Force Medical University, Xi’an, Shaanxi, China; ^3^ Skills Training Center, Xijing Hospital, Air Force Medical University, Xi’an, Shaanxi, China

**Keywords:** kidney transplantation, T cell-mediated rejection, M1-type macrophages, fluorescent probe, rejection reaction monitoring

## Abstract

**Background:**

T cell-mediated rejection (TCMR) represents a leading cause of graft dysfunction and even patient mortality following transplantation. Percutaneous biopsy for monitoring T-cell-mediated rejection (TCMR) presents several inherent limitations, including its invasive nature, the risk of procedure-related infections, potential iatrogenic injury to the graft kidney, and issues related to delayed monitoring. This study seeks to identify novel monitoring modalities to achieve early, non-invasive, dynamic monitoring of allograft rejection.

**Methods:**

The transplanted kidneys of Wistar-SD allogeneic kidney transplantation rats were analyzed by pathological methods and single-cell sequencing technology to identify the upregulated targets when rejection occurs. Based on these targets, a library was constructed and screened to obtain fluorescent probes for specific monitoring of rejection. After completing the safety verification of the probes, flow cytometry and *in vivo* imaging technology were used to verify the monitoring effect of the probes on rejection *in vitro* and *in vivo*, respectively.

**Results:**

In this study, we rationally developed a near-infrared fluorescent probe, XJYZ, for the *in vivo* imaging of M1 macrophages. We evaluated the capability of XJYZ for the early monitoring of rejection in an allogeneic renal transplantation model. *In vivo* imaging demonstrated that XJYZ preferentially accumulated within the allograft, enabling the early detection of dynamic changes in M1 macrophage infiltration.

**Conclusions:**

M1-type macrophages are recruited in large numbers in the early stage of transplantation and play a key role in the progression of rejection. Glucose transporter-1 (GLUT1) is crucial for M1-type macrophages to exert pro-inflammatory effects. In the early stage of rejection, due to the high metabolic demand of M1-type macrophages, the expression of GLUT1 is significantly upregulated. These findings highlight the potential of GLUT1 as a predictive biomarker for guiding early and precise monitoring of rejection. In conclusion, this study provides an alternative method for early and non-invasive monitoring of allograft rejection.

## Introduction

Renal transplantation is one of the most effective treatment strategies for end-stage renal disease (1). Despite the advances in surgical techniques and the maturation of clinical immunosuppressive regimens, which have significantly prolonged the survival of transplanted kidneys and patients, allogeneic transplantation rejection remains a major challenge to the long-term graft survival (2, 3).

T cell-mediated rejection (TCMR) is the most common type of rejection in the early post-transplant period following renal transplantation, accounting for approximately 90% of acute rejection episodes. It constitutes an independent risk factor for early graft dysfunction and is histopathologically characterized by T cell and macrophage recruitment and infiltration (4). At present, the “gold standard” for clinical diagnosis of TCMR is histological examination of renal biopsy samples (5). T cell-mediated rejection (TCMR) is histopathologically characterized by extensive T cell infiltration into the allograft, resulting in tubulointerstitial inflammation and/or arteritis (6). However, percutaneous biopsy carries inherent limitations, including its invasive nature, risk of infection, high cost, delayed diagnostic capability, limited repeatability within short timeframes, and provision of only static and localized pathological data (7, 8). Therefore, there is an urgent need to develop a novel approach for early, non-invasive and dynamic monitoring of transplantation rejection, so as to complement the conventional methods for monitoring rejection after kidney transplantation. Studies indicate that extensive infiltration of M1 macrophages in the early post-transplant phase predicts the progression of severe rejection, and their abundance is positively correlated with poor renal allograft outcomes (9). Histopathological analysis of renal allograft tissue in a patient with acute TCMR revealed that macrophages constituted 32% to 60% of the infiltrating cellular population (10). Patients with acute TCMR often exhibit a significant increase in M1 macrophage infiltration, suggesting that early infiltration of M1 macrophages in the renal allograft may be strongly associated with the development of TCMR (11). Therefore, M1 macrophages could represent a promising therapeutic target for monitoring acute rejection post-transplantation.

In this study, we developed a near-infrared (NIR) fluorescent probe XJYZ based on GLUT1 targeting M1 macrophages. *In vitro* validation confirmed the probe’s specific labeling capability for M1 macrophages. *In vivo* experiments using a rat renal allograft model showed that compared with pathological biopsy of renal allografts, the probe could visualize the infiltration of M1 macrophages in renal allografts earlier and non-invasively, thereby indicating the occurrence of TCMR in transplanted kidneys earlier. Additionally, the probe exhibits excellent safety profiles *in vivo* and enables real-time assessment of therapeutic efficacy of pharmacological interventions This study provides a non-invasive and early monitoring method for TCMR following kidney transplantation.

## Materials and methods

### Ethics statement

Our study complied with all relevant ethical regulations. Animals were provided by the Laboratory Animal Welfare and Ethics Committee of Air Force Medical University (Approval No.KY20223099-1). All rats were maintained in the animal facility of the Department of Animal Experiments of the Air Force Medical University according to the Laboratory Animal Welfare and Ethics Committee of the Air Force Medical University. All animal experiments were performed in accordance with recommended guidelines.

### Animal models

Allogeneic rat renal transplantation models were established (n=10) using Wistar rats as donors and Sprague-Dawley (SD) rats as recipients, with body weights ranging from 150 to 300 grams Both donors and recipients underwent surgical procedures under anesthesia maintained with Tiletamine-Zolazepam (Zoletil 50^®^;0.1-0.12 ml/100g, im). Specific renal transplantation and perfusion operations were performed according to the protocol of the Department of Surgery, Johns Hopkins University School of Medicine (12). In brief, the surgical sequence involved: native left nephrectomy in the recipient followed by orthotopic transplantation of the Wistar donor kidney into the left retroperitoneal compartment. By performing end-to-end anastomosis between the transplant renal artery and the recipient’s renal artery stump, the transplant renal vein was reconstructed with the recipient’s renal vein using a sleeve anastomosis technique via a 1.36-mm internal diameter plastic conduit. Simultaneously, with urinary continuity reestablished via ureteroneocystostomy to the recipient bladder. A schematic of the surgical procedure is shown in **Supplementary Figure S1** of the Supplementary material. The perfusion of transplanted kidney after operation is shown in **Supplementary Figure S2A** of the Supplementary material, and uretero-bladder-replantation is shown in **Supplementary Figure S2B** of the Supplementary Material. After the completion of transplantation, B-ultrasound was performed to detect the blood perfusion of the transplanted kidney and the filling status of the bladder, as shown in **Supplementary Figures S3A–D** in the Supplementary material.

A syngeneic rat renal transplantation model was used as the control (n=10), with both donors and recipients being Sprague-Dawley (SD) rats weighing 150–300 g. The anesthesia and surgical procedures were identical to those described above. After transplantation, the survival of recipients was observed and recorded.

Sham-operated control rats underwent a midline laparotomy with sequential dissection through the abdominal wall layers to expose the renal fossae. Peri-renal adipose tissue was meticulously dissected to achieve complete renal mobilization, followed by 40 minutes of controlled renal exposure. Continuous intraperitoneal irrigation with warm saline (37 °C) was maintained throughout the exposure period to prevent tissue desiccation. The procedure concluded with multi-layer closure of the muscular planes and cutaneous suturing. The survival of rats after transplantation was observed and recorded. Infection prophylaxis was initiated immediately after surgery with intramuscular penicillin G (1ml/100g, im), followed by sustained analgesia through subcutaneous meloxicam administration (5 mg/kg, sc) to mitigate surgical stress responses.

### Histological staining and immunohistochemistry

Transplant recipients were euthanized(40% CO2, inh) at 24h, 48h, 72h, 120h, 7d, and 14d post-transplantation. Renal allograft tissues were fixed in formalin and embedded in paraffin. Serial sections (5 μm thickness) were prepared using a microtome. Paraffin-embedded sections were stained with hematoxylin and eosin (H&E). Histopathological evaluation was performed under standard light microscopy by two independent pathologists, each with over 5 years of experience. Renal allograft rejection was graded according to the Banff classification criteria (13).

Paraffin sections were stained with Masson staining, and images were examined using an optical microscope (E100, Nikon Instruments Inc.). Immunohistological labeling of sections was performed using polyclonal anti-CD86 antibody (ab220188, Abcam), anti-GLUT1 antibody (ab115730, Abcam), and CD3 antibody (ab11089, Abcam), with visualization achieved using corresponding secondary antibodies (ab6721, Abcam). Each specimen was randomly evaluated in three different fields of view.

### Preparation of single-cell suspension and scRNA-seq procedure

Subsets of rats (n=3) were euthanized on postoperative days 1, 2, and 3. Renal allografts were perfused via the aorta with ice-cold Dulbecco’s phosphate-buffered saline (DPBS) to eliminate erythrocytes prior to harvest. Kidneys were placed on ice-cold DPBS and bisected along the longitudinal axis. Cortical tissues were minced into fragments of approximately 1 mm³ using sterile scalpel blades until homogeneous disintegration was achieved. Tissue fragments underwent enzymatic digestion with collagenase IV (17104019, Gibco) to generate single-cell suspensions. Single-cell RNA sequencing libraries were constructed using the 10x Genomics platform (minimum sequencing depth: 50,000 reads per cell).

The raw single-cell sequencing data were aligned to the rat reference genome (Rnor_6.0) using Cell Ranger (v7.1.0). Quality control criteria included: retaining cells with 200–6000 detected genes, mitochondrial gene proportion <15%, and exclusion of erythrocytes and doublets. Integrative analysis was performed using the Seurat package (v5.0.1). Macrophages were identified by classic marker genes (Adgre1, CD68), and M1 subpopulations were defined by CD86 expression. The infiltration ratio of CD86+ macrophages was calculated from Seurat clustering results, and temporal dynamics were analyzed by ANOVA (p < 0.05 considered significant).

CellChat (version 1.0.0) was used to predict major signaling inputs and outputs of cells and how these signals coordinate functional cells and signaling via network analysis and pattern recognition, thereby parsing communications between M1 macrophages (Cd86+) and other immune cells. Chord diagrams were used for visualization to display specific interactions of M1 macrophages. Cell type annotation was performed using the SingleR (version 1.4.1) package. Violin plots and heatmaps of gene expression were generated using “VlnPlot” and “DoHeatmap,” respectively.

### Western blotting

Western blotting was performed to assess the protein expression levels of GLUT1.

Electrophoresis: An equal amount of protein (20-30 μg per lane) from each sample was mixed with 5x Laemmli loading buffer, denatured at 95 °C for 5 min, and then separated by 10% sodium dodecyl sulfate-polyacrylamide gel electrophoresis (SDS-PAGE).Transfer: The separated proteins were electrophoretically transferred onto a polyvinylidene difluoride (PVDF) membrane using a wet transfer system at 100 V for 60–90 min on ice. Blocking: The membrane was blocked with 5% (w/v) non-fat dry milk or BSA in Tris-Buffered Saline containing 0.1% Tween-20 (TBST) for 1 hour at room temperature to prevent non-specific binding. Primary antibody incubation: Incubate the membrane at 4 °C overnight. During this period, the following diluted primary antibodies were used for reaction with it. These antibodies were all dissolved in blocking buffer: rabbit anti-GLUT1 antibody (ab115730, Abcam), GAPDH (GB15003, Servicebio). Washing: The membrane was washed three times for 10 min each with TBST. Secondary antibody incubation: Subsequently, the membrane was incubated with the corresponding horseradish peroxidase (HRP)-labeled secondary antibodies at room temperature for 1 hour: goat anti-rabbit IgG against GLUT1 and goat anti-rabbit IgG against GAPDH. Washing: The membrane was washed again three times for 10 min each with TBST. Detection: Protein bands were visualized using an enhanced chemiluminescence (ECL) detection kit. The chemiluminescent signals were captured and analyzed using a chemiluminescence imaging system (SCG-W3000, Servicebio).

### Cell culture

DMEM high-glucose medium (11965092) and fetal bovine serum (A5256701) were procured from Gibco. Six-to-eight-week-old mice were euthanized by cervical dislocation, followed by 5-minute disinfection in 75% ethanol. Under aseptic conditions, femurs and tibiae were isolated with attached muscle tissues excised. Bone ends were resected, and bone marrow was flushed into centrifuge tubes using ice-cold phosphate-buffered saline (PBS) via a 1 mL syringe fitted with a 25-gauge needle. Flushing was repeated until the marrow cavity appeared pale. The cell suspension was centrifuged (300 ×g, 5 min, 4 °C), then resuspended in 2 mL erythrocyte lysis buffer (C3702, Beyotime) for 3-minute incubation at room temperature (strictly timed to prevent over-digestion). Lysis was terminated by adding 10 mL PBS, followed by centrifugation and supernatant removal. Cells were resuspended in complete medium supplemented with 20 ng/mL macrophage colony-stimulating factor (M-CSF; 315-02-10UG, Gibco), counted, and plated at 1×10^6^ cells/mL. Cultures were maintained at 37 °C with 5% CO_2_, with half-medium replacement performed on day 3 to remove non-adherent cells. M0 macrophages were obtained by days 6–7 of differentiation.

Resting M0 macrophages were polarized into M1 or M2 phenotypes by stimulating with 100 ng/mL LPS (00-4976-93, Gibco) and 20 ng/mL IFN-γ (315-05-500UG, Gibco) for 24 hours, or 40 ng/mL IL-4 (214-14-20UG, Gibco) and 40 ng/mL IL-13 (210-13-10UG, Gibco) for 48 hours.

### Immunofluorescence staining and confocal microscopy experiment

Immunofluorescence staining was used to characterize the number of M1 macrophages and GLUT1 expression in transplanted kidneys. Anti-rat CD86 antibody (ab213045, Abcam) and anti-rat GLUT1 antibody (ab115730, Abcam) were combined with Alexa Fluor 488-conjugated goat anti-mouse IgG (1:400, GB25301, Servicebio) secondary antibody, and DAPI (G1012, Servicebio) was added. Images were captured using an upright fluorescence microscope (Nikon Eclipse C1, Nikon, Japan).

Confocal microscopy was employed to investigate the targeting efficacy of probe XJYZ on M0, M1, and M2 macrophages. Bone marrow-derived primary cells were isolated and subsequently stimulated to differentiate into M0, M1, and M2 macrophages. These macrophage subtypes were then incubated with probe XJYZ for 24 hours. After washing off the unbound probe, cells were allowed to adhere to coverslips for 24 hours. Subsequently, the cells were fixed with 4% paraformaldehyde solution (G1101-3ML, Servicebio). Following fixation, cell nuclei were stained with DAPI (G1012, Servicebio). Images were acquired using an inverted microscope (NIKON Eclipse Ti, Nikon, Tokyo, Japan). The imaging channels used were as follows: DAPI (excitation wavelength 405 nm, emission wavelength 417–477 nm); and XJYZ (excitation wavelength 622 nm, emission wavelength 570–1000 nm).

### Library design and high-throughput screening

The library was composed of carbohydrate scaffolds from 10 β-D-pyranose glucose analogs combined with 3 common fluorescent compounds, randomly assembled into 30 compounds (**Supplementary Figure 9**). Fluorescent moieties in the library were purchased from MedChemExpress (New Jersey, USA), and carbohydrate scaffolds were obtained from Merck (Darmstadt, Germany).

For cell screening, M0, M1, and M2 macrophages were seeded into 96-well plates and incubated with 0.5 μM library at 37 °C for 1 hour. After washing with PBS, fluorescence intensity was measured using a microplate reader. Corrected fluorescence values were calculated by subtracting blank group values from sample values. Relative fluorescence expression was determined by normalizing M1-corrected values to those of M0 and M2 groups.

### Flow cytometry

Verification of Macrophage Polarization: M0 macrophages were incubated with FITC-conjugated anti-mouse F4/80 antibody (52267, Cell Signaling Technology) for 1 hour at 4 °C in the dark. M1 macrophages were incubated with FITC-conjugated anti-mouse F4/80 antibody (52267, Cell Signaling Technology) and APC-CY7-conjugated anti-mouse CD86 antibody (A17199A, Biolegend) for 1 hour at 4°C in the dark. M2 macrophages were incubated with FITC-conjugated anti-mouse F4/80 antibody (52267, Cell Signaling Technology) and PE-conjugated anti-mouse CD206 antibody (PE-98031, Proteintech) for 1 hour at 4°C in the dark. Cells were subsequently washed with phosphate-buffered saline (PBS) and analyzed by flow cytometry (BD FACSCelesta). Data were processed and analyzed using Flowjo software.

Verification of Probe Targeting Specificity to Macrophage Subtypes: M0 macrophages were incubated with probe XJYZ and FITC-conjugated anti-mouse F4/80 antibody (52267, Cell Signaling Technology) for 1 hour at 4°C in the dark. M1 macrophages were incubated with probe XJYZ and APC-conjugated anti-mouse CD86 antibody (84393, Cell Signaling Technology) for 1 hour at 4°C in the dark. M2 macrophages were incubated with probe XJYZ and PE-conjugated anti-mouse CD206 antibody (PE-98031, Proteintech) for 1 hour at 4°C in the dark. After incubation, cells were washed with phosphate-buffered saline (PBS) and analyzed by flow cytometry (Cytomics FC 500, Beckman Coulter). Data were processed and analyzed using Flowjo software.

### Biosafety evaluation

The cytotoxicity of XJYZ was evaluated using a CCK-8 kit M0, M1, and M2 macrophages were seeded into 96-well plates at a density of 5×10³ cells per well and incubated with 0, 0.1, 1.0, 10, and 100 μM XJYZ for 24 hours at 37°C in 5% CO_2_. Each test concentration was performed in triplicate. After incubation, cells were washed twice with PBS, and 10 μL of CCK-8 reagent was added to each well for an additional 1-hour incubation. Optical density (OD) values were measured at 450 nm using a Varioskan ALF multimode microplate reader (VA000010C, Thermo Scientific) Flow cytometry was employed to assess apoptosis in cells following co-incubation with the probe. After incubating M0, M1, and M2 macrophages separately with 1μM probe XJYZ for 24 hours, cells were stained using an Annexin V-FITC Apoptosis Detection Kit (C1062S, Beyotime). Apoptosis was then quantified by flow cytometry (Cytomics FC 500, Beckman Coulter) to further evaluate the cytotoxicity of probe XJYZ.

For *in vivo* safety validation, healthy Sprague-Dawley rats (200 ± 20 g) received intravenous administration of 1 mM probe XJYZ via the tail vein. Control cohorts were injected with an equal volume of phosphate-buffered saline (PBS) using identical delivery parameters. On days 1, 3, and 7 post-administration, rats from both the experimental and control groups were sacrificed for necropsy, and major organs (heart, liver, spleen, lungs, and kidneys) were systematically collected for histopathological examination. Venous blood samples were collected for comprehensive biochemical profiling, including measurement of alanine aminotransferase (ALT), aspartate aminotransferase (AST), creatine kinase (CK), lactate dehydrogenase (LDH), creatinine (CREA), and urea (UREA), alongside complete blood count analysis encompassing white blood cells (WBC), erythrocytes (RBC), and platelets (PLT).

### Quantitative PCR

Real-time quantitative PCR (RT-qPCR) was performed to determine the mRNA expression levels of M1 macrophages and GLUT1 in transplanted kidneys. Briefly, total mRNA was extracted and reverse-transcribed according to the manufacturer’s instructions. Subsequently, cDNA amplification was performed using a polymerase chain reaction thermal cycler (A24811, Thermo Scientific) with the following cycling conditions: 10 minutes at 95°C, followed by 40 cycles of 15 seconds at 95°C and 1 minute at 55°C. Each experiment was independently repeated three times. Quantitative analysis was normalized to GAPDH. Primers used are listed in **Supplementary Table 2**.

To validate successful macrophage polarization, mRNA expression levels of M1and M2 associated markers were measured *in vitro* using the same protocol described above. Primers sequences are provided in **Supplementary Table 2**.

### *In vivo* imaging and fluorescence quantification

Syngeneic and allogeneic renal transplant rats were administered XJYZ (1 mM) via tail vein injection. Following probe administration, fluorescence imaging was performed using a PerkinElmer IVIS Lumina III system (excitation/emission wavelength = 622 nm/663 nm) with a 5-second acquisition time. Subsequently, allogeneic renal transplant rats were sacrificed in batches at different time points, and transplanted kidneys along with major organs were harvested for ex vivo imaging. Fluorescence intensity in regions of interest (ROIs) was quantitatively analyzed using Spectrum Living Image 4.0 software.

### Statistical analysis

Quantitative results are presented as mean ± standard deviation (SD). For statistical comparisons involving more than two experimental groups, one-way analysis of variance (ANOVA) was used. Unpaired t-tests were applied for comparisons between two data sets. Statistical significance was defined as a p-value < 0.05.

## Result

### Establishment of rat renal transplantation model

**Figure 1:**
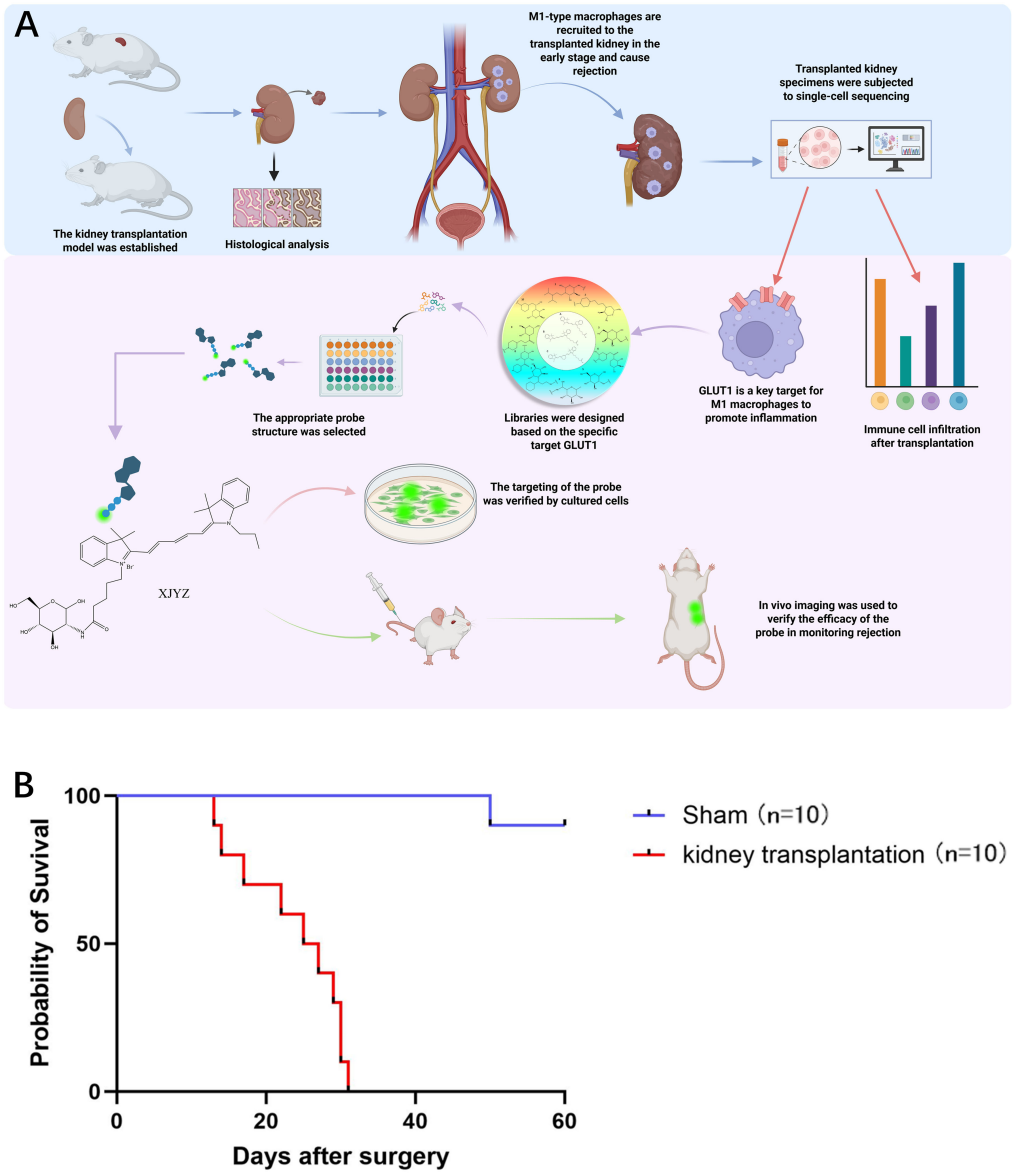
Design process of the probe targeting M1 macrophages and survival status in allogeneic rat kidney transplantation. **(A)** Following the establishment of an allogeneic rat kidney transplantation model, histological analysis and single-cell sequencing revealed that M1 macrophages infiltrated the transplanted kidney in large numbers during the early post-transplant period. Single-cell sequencing further demonstrated that GLUT1 is a critical target involved in the pro-inflammatory function of M1 macrophages within the transplanted kidney, contributing to the initiation of rejection. Based on GLUT1, we synthesized a probe specifically targeting M1 macrophages and validated its targeting efficacy and ability to monitor rejection both *in vitro* and *in vivo*. **(B)** Survival status of Wistar-SD rats following kidney transplantation compared with sham surgery (n = 10).

As shown in **Figure 1A**, this diagram illustrates our overall experimental workflow.

Ten successful renal transplantations were performed in the Wistar-SD allogeneic rat model, with all graft recipients surviving beyond postoperative day 3 (POD3), thereby excluding procedure-related mortality. The maximum graft survival duration reached 32 days (**Figure 1B**). Doppler ultrasonography performed on POD1 demonstrated patent blood perfusion in renal allografts (**Supplementary Figure 3A**), unobstructed venous outflow (**Supplementary Figure 3B**), and physiological bladder distension (**Supplementary Figure 3D**). In the sham-operated control cohort (n=10 SD rats), one mortality occurred at POD51 attributable to anastomotic infection, while the remaining nine animals survived >60 days (**Figure 1B**).

### Increased infiltration of M1 macrophages in allogeneic renal allografts

Following kidney transplantation in Wistar-SD rats, selected recipients were euthanized at various time points postoperatively for histological evaluation.

**Figure 2:**
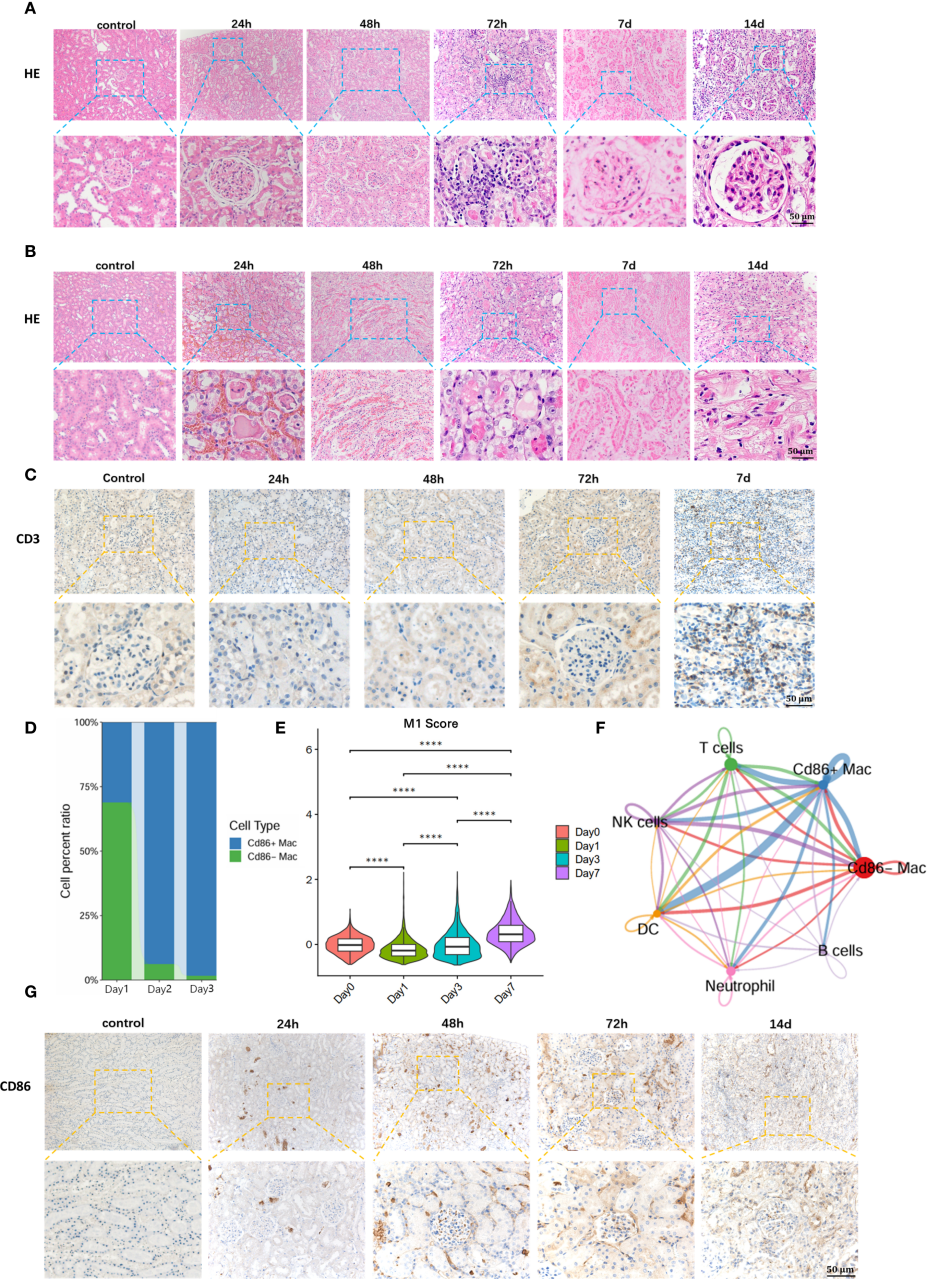
Early detection of M1 macrophages is of significant importance for early warning of T-cell recruitment and diagnosis of TCMR. **(A)** Hematoxylin and Eosin (H&E) staining of renal allograft cortex. **(B)** H&E staining of renal allograft medulla. **(C)**T cell recruitment in renal allograft. **(D)** Single-cell sequencing reveals massive infiltration of M1 macrophages in early post-transplantation period. **(E)** Temporal dynamics of the M1 macrophage-specific inflammatory effector gene set during the early post-transplantation phase. **(F)** Communication between M1 macrophages and T cells in renal allograft. **(G)** Immunohistochemical staining for anti-CD86 validates M1 macrophage infiltration in renal allograft. ****:P<0.0001.

Histopathological alterations characteristic of TCMR were observed in the allograft kidneys over time. Within 48 hours post-transplantation, cortical endothelial cell proliferation and mesangial cell hyperplasia were noted, with enlargement of the glomerular tufts and narrowing of Capsular space. Concurrently, significant tubular injury in the medulla was evident as early as 24 hours, characterized by the presence of proteinaceous casts, marked edema, and vacuolar degeneration of tubular epith cellselial. By 48 hours, the lesions progressed to extensive interstitial hemorrhage and features of acute tubular necrosis, including necrotic debris within the tubular lumens, sloughing of epithelial cells, indistinct tubular architecture, and severe granular and vacuolar degeneration of tubular epithelial cells (**Figures 2A, B**). The severe damage observed in the transplanted kidneys suggests the onset of rejection.

Key inflammatory features of TCMR were most prominent at 72 hours. Marked interstitial inflammatory cell infiltration was observed, accompanied by endothelial and mesangial cell proliferation. Critically, tubulitis - a hallmark of TCMR - was evident in the renal medulla, with inflammatory cells infiltrating individual renal tubule cross-sections Medullary changes also included hyaline casts, tubular atrophy, hypertrophy of tubular epithelial cells with cytoplasmic eosinophilia, and karyopyknosis (**Figures 2A, B**) According to Banff criteria (13), significant interstitial inflammation (i=2) and tubulitis (t=1) were observed at 72 hours post-transplant, consistent with the diagnosis of TCMR.

On postoperative day 7 (POD 7), the pathological changes further intensified. The glomeruli exhibited severe fibrosis with indistinct Capsular space architecture. The renal tubules demonstrated widespread atrophy and necrosis, with obliteration of discernible lumen structures and accumulation of necrotic debris and sloughed cells within the lumens. By postoperative day 14 (POD 14), the glomeruli manifested severe capillary loop atrophy, and the interstitium showed extensive fibrosis (**Figures 2A, B**).

To further define the time points of T cell infiltration in transplanted kidneys with TCMR, immunohistochemistry for anti-CD3+ T cells was performed on recipient specimens. Results showed extensive infiltration of T cells in renal allografts at POD 7(**Figure 2C**). Compared with the appearance of typical pathological changes in TCMR, including tubulitis and interstitial inflammation at day 3 (D3), significant T cell infiltration occurred later, suggesting that diagnosis targeting T cells for TCMR may have a lag phase.

To identify early diagnostic biomarkers for TMCR, single-cell RNA sequencing was performed on renal allograft specimens from allogeneic rat recipients at serial timepoints. Analysis revealed progressive recruitment of M1 macrophages (CD86-Positive Macrophages) commencing at 24 hours post-transplantation, with significant proportional expansion peaking at 72h (**Figure 2D**). We selected and monitored the gene set associated with the pro-inflammatory effects in M1 macrophages, observing that the inflammatory genes exhibited an increasing trend during the early post-transplantation period (**Figure 2E**).In contrast, maximal T cell infiltration occurred at POD7. These findings demonstrate predominant early-phase infiltration of M1 macrophages preceding adaptive immune cell recruitment.

Subsequent Intercellular communication analysis demonstrated a statistically significant correlation between M1 macrophage infiltration and T cell recruitment (**Figure 2F**, **Supplementary Figure 6**). This was in accordance with the previous reports that M1 macrophages release pro-inflammatory factors such as TNF-α (tumor necrosis factor-α), IL-6 (interleukin-6), and IL-12 (interleukin-12), activating the NF-κB signaling pathway. This leads to endothelial and tissue cells expressing chemokines and adhesion molecules, thereby mediating T cell recruitment to the graft site (14). Additionally, immunohistochemical staining for anti-CD86 positive targets was performed on renal allograft specimens to further validate M1 macrophage infiltration. The immunohistochemical results were consistent with the sequencing data, showing significant M1 macrophage infiltration in the early post-transplantation period, particularly at 72 hours (**Figure 2G**). Collectively, these findings indicate that M1 macrophage influx precedes both T cell infiltration and histopathological manifestations in rat renal allografts, suggesting their potential utility as early detectors of incipient T cell-mediated rejection.

### GLUT1 is highly expressed in M1 macrophages

Given that M1 macrophages predominantly utilize glucose transporter-1 (GLUT1) to facilitate carbohydrate uptake sustaining aerobic glycolysis (15), their heightened energy demands during proinflammatory activation necessitate GLUT1 upregulation. Consequently, monitoring GLUT1 expression dynamics serves as a critical strategy for spatiotemporal quantification of M1-polarized macrophage infiltration.

**Figure 3:**
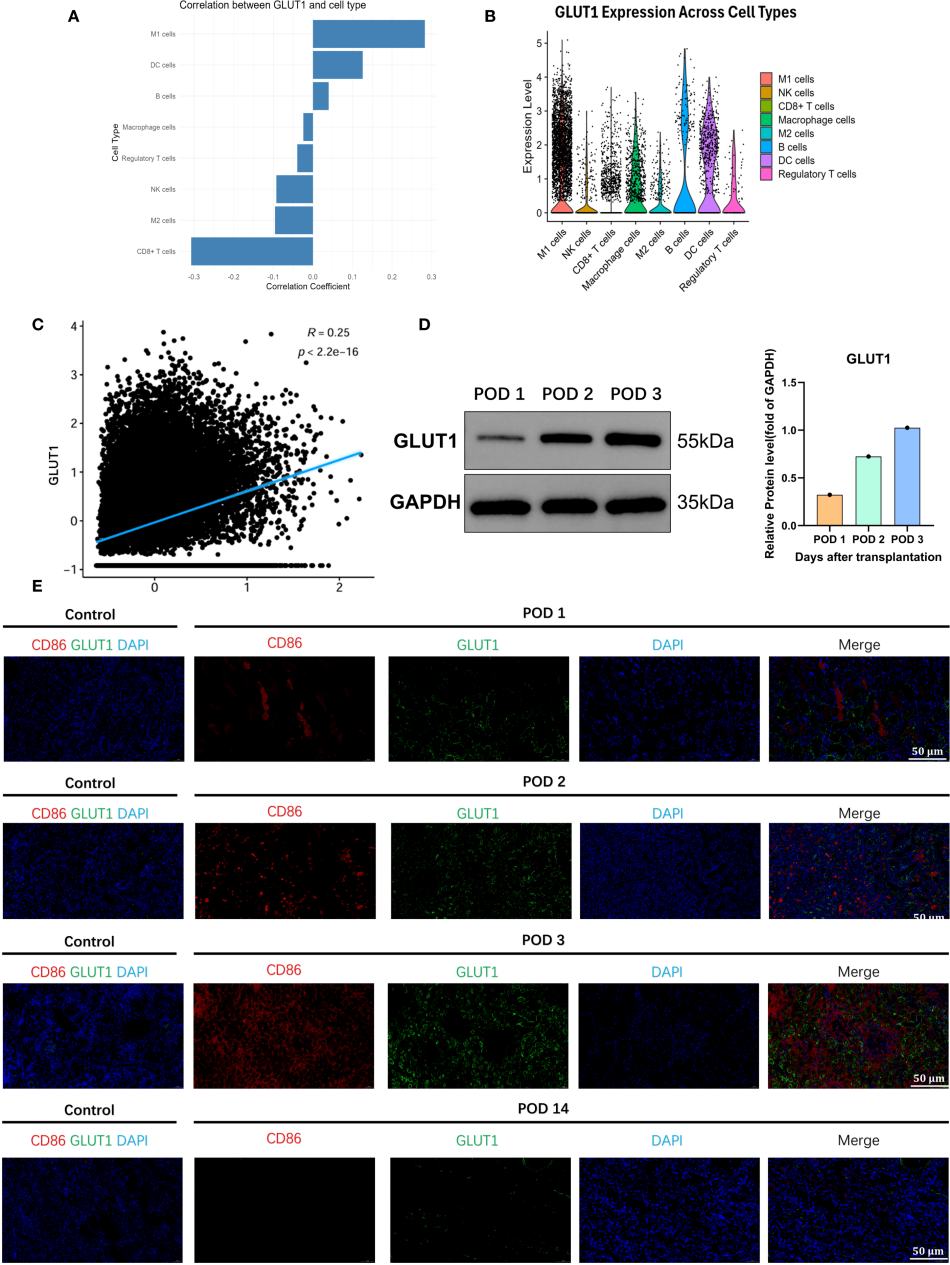
GLUT1 as the pivotal metabolic regulator of proinflammatory effector functions in M1-polarized macrophages. **(A)** GLUT1 is highly correlated with M1 macrophages infiltrating the transplanted kidney. **(B)** Correlation of GLUT1 with various immune cells in the transplanted kidney. **(C)** Association between the inflammatory-related gene set of M1 macrophages and GLUT1. **(D)** The expression level of GLUT1 protein in the transplanted kidney. **(E)** Co-localization of GLUT1 with M1 macrophages in the transplanted kidney.

To validate whether GLUT1 is upregulated in renal allografts with acute rejection, we reviewed the sc-seq data of the kidney allograft 72 hours posttransplant and found that GLUT1 was highly correlated with M1 macrophages in the graft (**Figure 3A**). GLUT1 was predominantly expressed in M1 macrophages, with low expression in unpolarized M0 or anti-inflammatory M2 macrophages and other immune cells (**Figure 3B**, **Supplementary Figure 7**). A high correlation was observed between the expression of inflammatory genes in M1 macrophages and GLUT1 expression (**Figure 3C**). Subsequently, we examined GLUT1 protein expression in the transplanted kidney specimens, and the results demonstrated that its expression exhibited an increasing trend over time in a time-dependent manner (**Figure 3D**).We further colocalized immunofluorescence colocalization of CD86 and GLUT1 on renal allograft specimens, further confirming that M1 macrophages and GLUT1 were widely expressed in the early post-transplant period, with both reaching peak expression at 72h (**Figure 3E**). These results indicate that GLUT1 is upregulated in allogeneic rat renal transplantation with TCMR, predominantly expressed in M1 macrophages, and may serve as a monitoring target.

### A probe (XJYZ) specifically targeting M1 macrophages was designed and screened from a library based on GLUT1

**Figure 4:**
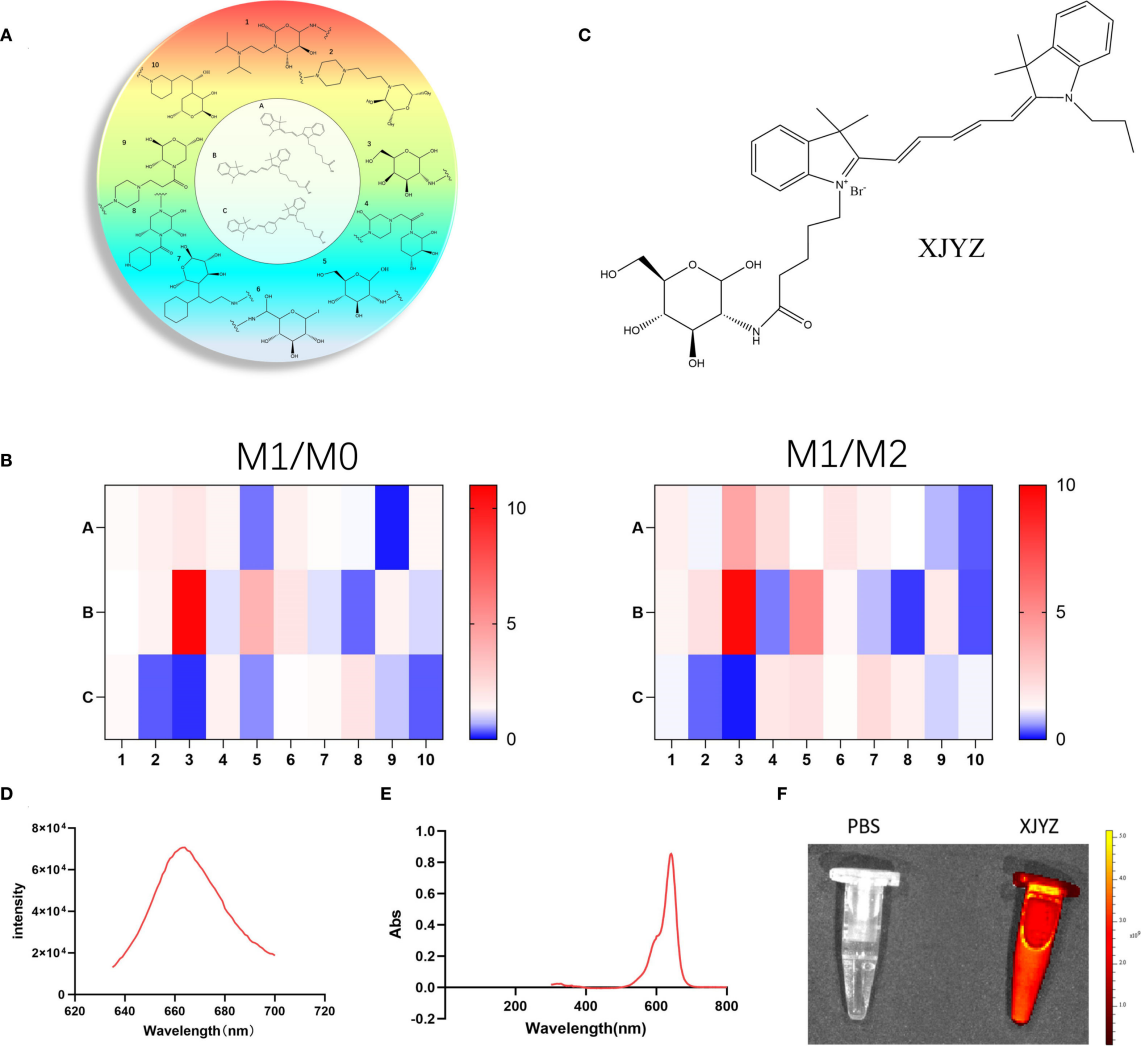
Design of a GLUT1-related library and screening for a probe (XJYZ) specifically targeting M1 macrophages. **(A)** Design of GLUT1-based library. **(B)** Screening of probes with specific targeting to M1 macrophages after co-culture with M0, M1, and M2 macrophages. **(C)** Structure of probe XJYZ. **(D)** Fluorescence excitation spectrum of probe XJYZ. **(E)** UV-Vis absorption spectrum of probe XJYZ. **(F)** Fluorescence imaging of probe XJYZ obtained by PerkinElmer IVIS imaging system (λex/em = 622/663 nm).

The primary approach for developing probes with specific targeting ability is library-based screening. We designed a library based on GLUT1 to screen for probe structures with the highest specificity for M1 macrophages. A library was constructed featuring ten carbohydrate backbones conjugated with three fluorescent moieties (**Figure 4A**), including common fluorophores CY3, CY5, and CY7 (**Supplementary Figure 10**). Given that GLUT1 primarily uptakes β-D-pyranose glucose, carbohydrate scaffolds were designed by mimicking the structure of β-D-pyranose glucose (**Supplementary Figure 11**).

To screen for probe structures specifically targeting M1 macrophages, bone marrow-derived macrophages (BMDMs) isolated from C57BL/6 mice were polarized *in vitro* and co-incubated with combinatorial library compounds (1 μM) Probe selectivity was quantified via the M1/M2 selectivity index (SI), with results visualized in a heatmap (**Figure 4B**). This screening identified XJYZ as the optimal probe demonstrating superior targeting specificity toward M1-polarized macrophages (**Figure 4C**). Structurally, XJYZ comprises a cyanine dye CY5 fluorophore conjugated to a β-D-glucopyranose analogue through a C2-position amide linkage. This finding is consistent with previous literature reports that M1 macrophages have a high affinity for the CY5 fluorophore.

The targeted action of probe XJYZ toward M1 macrophages is mediated by two structural components: initially, the hydrophobic groups, such as multiple aromatic rings and a long carbon chain contained within the CY5 fluorophore, contribute to its phagocytosis by macrophages, and subsequently, leveraging the enhanced glycolytic capacity of M1 macrophages, the β-D-glucopyranose analog structure is specifically internalized by M1 macrophages to achieve precise targeting.

The synthetic protocol of probe XJYZ is described in **Supplementary Scheme 1**. The structure of the probe was characterized by 1H NMR and ESI-MS (**Supplementary Figure 26, 27**). We explored the fluorescence response of probe XJYZ, which exhibited a characteristic absorption peak at 663 nm under excitation at 622 nm (**Figure 4D**). The emission band spanned from 640 to 695 nm, closely matching the NIR-I window. The probe demonstrated a gradual decay trend on the long-wavelength side (>680 nm), with 26.5% intensity retention at 700 nm, indicating significant luminescence capacity in the deep tissue penetration wavelength range. Subsequently, we performed UV absorption characterization and observed a prominent absorption peak at 641–642 nm (maximum absorbance of 0.854), which is characteristic of the main electronic transition of the probe molecule and consistent with the absorption properties of NIR-I fluorophores. The absorption peak exhibited a symmetrical distribution with a full width at half maximum (FWHM) of approximately 50 nm, confirming a clear electronic transition process. The absorbance formed a plateau near the peak (640–650 nm) and rapidly decreased to baseline levels after 700 nm. Additionally, the absorbance approached zero (Abs < 0.005) at wavelengths below 500 nm or above 750 nm, verifying minimal background interference for probe XJYZ within the NIR-I window (**Figure 4E**). Combining the results of fluorescence excitation and UV absorption characterization, a 22-nm Stokes shift was observed between the emission peak and the main absorption peak of XJYZ, which can effectively reduce self-absorption interference and enhance the signal-to-noise ratio in *in vivo* imaging. Fluorescence imaging using the PerkinElmer IVIS imaging system demonstrated the emission capability of XJYZ (**Figure 4F**).

### The probe XJYZ exhibits excellent targeting specificity towards M1 macrophages *in vitro*

**Figure 5:**
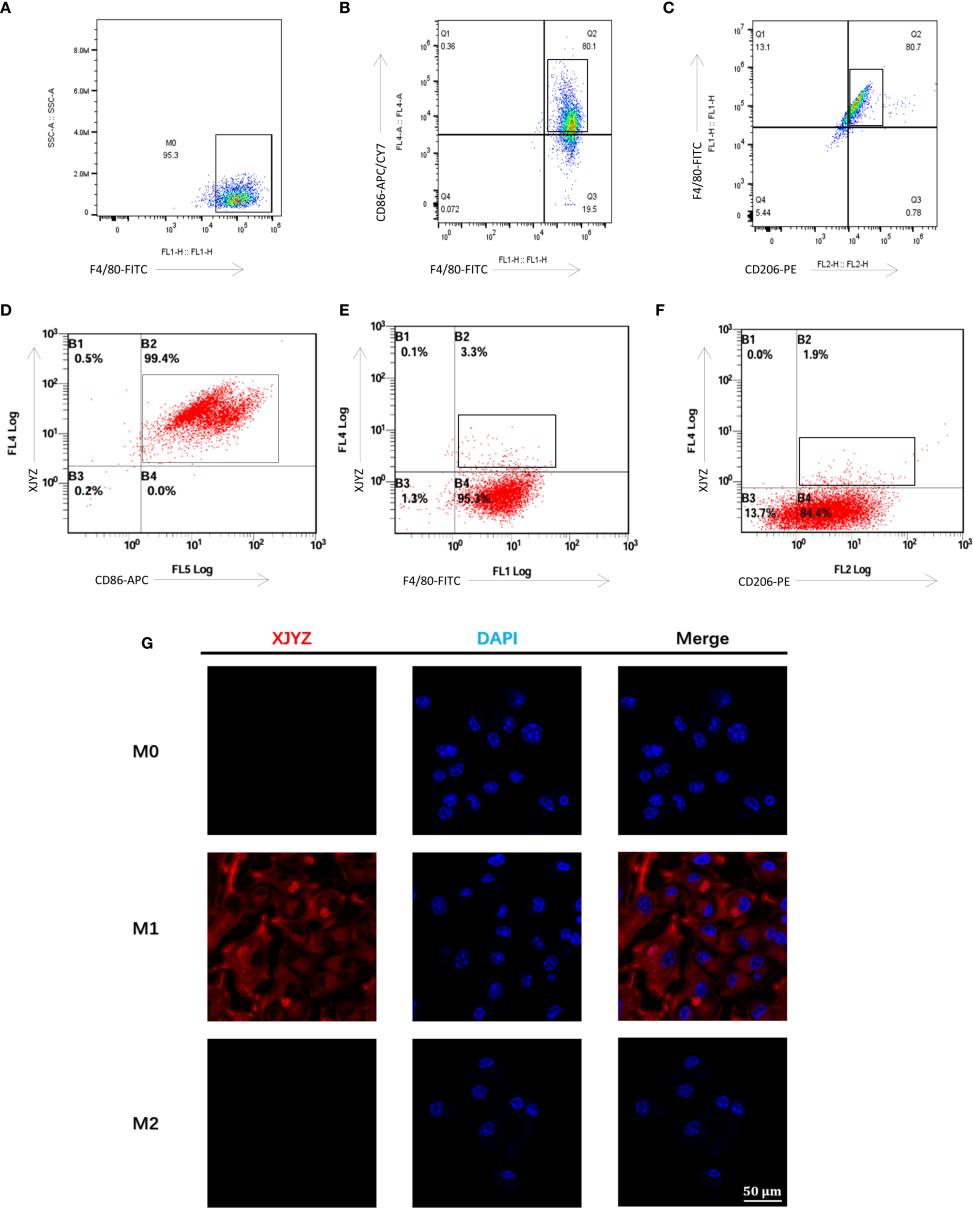
Cellular targeting validation of the probe XJYZ. **(A)** Flow cytometry validation of successful polarization of M0 macrophages. **(B)** Flow cytometry validation of successful polarization of M1 macrophages. **(C)** Flow cytometry validation of successful polarization of M2 macrophages. **(D)** Flow cytometry validation of specific targeting of probe XJYZ to M1 macrophages. **(E)** Flow cytometry validation of no specific targeting of probe XJYZ to M0 macrophages. **(F)** Flow cytometry validation confirming the absence of specific targeting of probe XJYZ to M2 macrophages. **(G)** Confocal microscopy validation of targeting of XJYZ to M1 macrophages.

To further validate the targeting specificity of probe XJYZ, BMDM (Bone Marrow-Derived Macrophages) were re-isolated (**Supplementary Figure 12**) and subjected to *in vitro* polarization, with successful polarization confirmed by flow cytometry (**Figures 5A–C**).

Probe XJYZ and anti-CD86 antibody were used for co-labeling of M1 macrophages to validate XJYZ targeting, showing a double-positive rate of 99.7% (**Figure 5D**), which confirmed the highly specific binding capacity of XJYZ to M1 macrophages *in vitro*. To determine whether XJYZ targets M0/M2 macrophages, co-labeling with anti-F4/80 (for M0) or anti-CD206 (for M2) was performed: only 3.3% of M0 cells were double-positive for XJYZ and F4/80, with 95.3% singly labeled by F4/80 (**Figure 5E**); similarly, only 1.9% of M2 cells were double-positive for XJYZ and CD206, with 84.4% singly labeled by CD206 (**Figure 5F**). These results demonstrated that XJYZ exhibits no specific targeting to M0/M2 macrophages *in vitro*, with exclusive specificity for M1 macrophages.

Confocal microscopy analysis demonstrated significant binding of probe XJYZ to M1 macrophages, while negligible targeting was observed toward M0 or M2 phenotypes (**Figure 5G**), substantiating the probe’s discriminatory capacity among macrophage polarization states.

### The probe XJYZ does not induce cytotoxicity or toxicity of tissue

**Figure 6:**
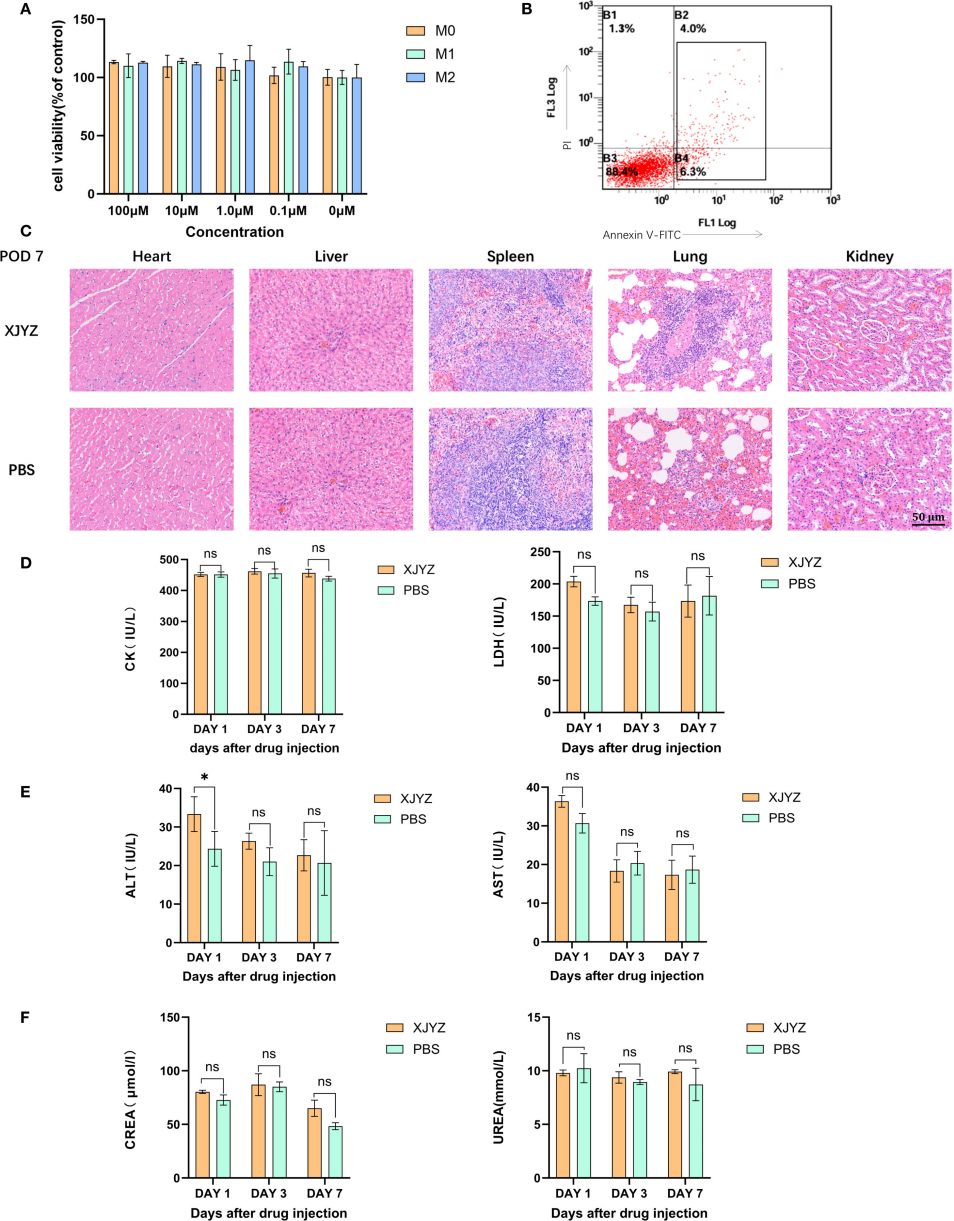
*In vivo* and *in vitro* safety validation of probe XJYZ. **(A)** *In vitro* safety profile of probe XJYZ assessed by CCK-8 assay. **(B)** Apoptosis assay following co-incubation of the probes with cells. **(C)** Hematoxylin and eosin (H&E) staining of major organs harvested 7 days post-intravenous injection via tail vein. **(D)** Biochemical analyses of biomarkers for cardiac. **(E)** Biochemical analyses of biomarkers for hepatic. **(F)** Biochemical analyses of biomarkers for renal. All control groups received intravenous administration of an equivalent volume of phosphate-buffered saline (PBS). Data in Figure **(A–E)** are expressed as mean ± standard deviation; ns denotes no statistically significant difference. *:P<0.05.

To validate the cytotoxicity of XJYZ, four concentration gradients (0.1μM, 1.0 μM, 10 μM, 100 μM) of probe XJYZ were co-incubated with M0, M1, and M2 macrophages. PBS was added to control cells, and the CCK8 assay was used to verify the effect of the probe on cell viability *in vitro*. The results showed that probe XJYZ did not significantly affect cell survival (**Figure 6A**, **Supplementary Figure 16**). Furthermore, after co-incubation of 1 μM XJYZ with M1 macrophages, Annexin V-FITC/PI dual staining revealed that apoptotic cells accounted for 10.4% of the total cell population, indicating an acceptable level of cytotoxicity of the probe (**Figure 6B**, **Supplementary Figure 17**).

In *in vivo* experiments, SD rats in the administration group were injected with 1 mM probe XJYZ via the tail vein, while SD rats in the control group were injected with an equal volume of PBS via the tail vein. On days 1, 3, and 7 post-injection, major organs (heart, liver, spleen, lungs, and kidneys) were collected from both the drug-administered group and the control group. No significant pathological alterations were observed in the major organs of the drug-administered group. (**Figure 6C**, **Supplementary Figure 18**).

In addition, we assessed biochemical markers reflective of cardiac function (creatine kinase and lactate dehydrogenase), hepatic function (alanine aminotransferase and aspartate aminotransferase), and renal function (urea and creatinine) to further evaluate the safety profile of XJYZ. The results indicated that administration of probe XJYZ did not impair cardiac, hepatic, or renal function (**Figures 6D–F**). Furthermore, hematological parameters, including white blood cells, red blood cells, platelets, hemoglobin, mean corpuscular hemoglobin, mean corpuscular hemoglobin concentration, and platelet distribution width, were all within the normal range in both the XJYZ-treated group and the control group (**Supplementary Table S1**). These findings collectively confirmed the excellent biocompatibility of XJYZ.

### Probe XJYZ for early warning of TCMR in rat renal transplantation

**Figure 7:**
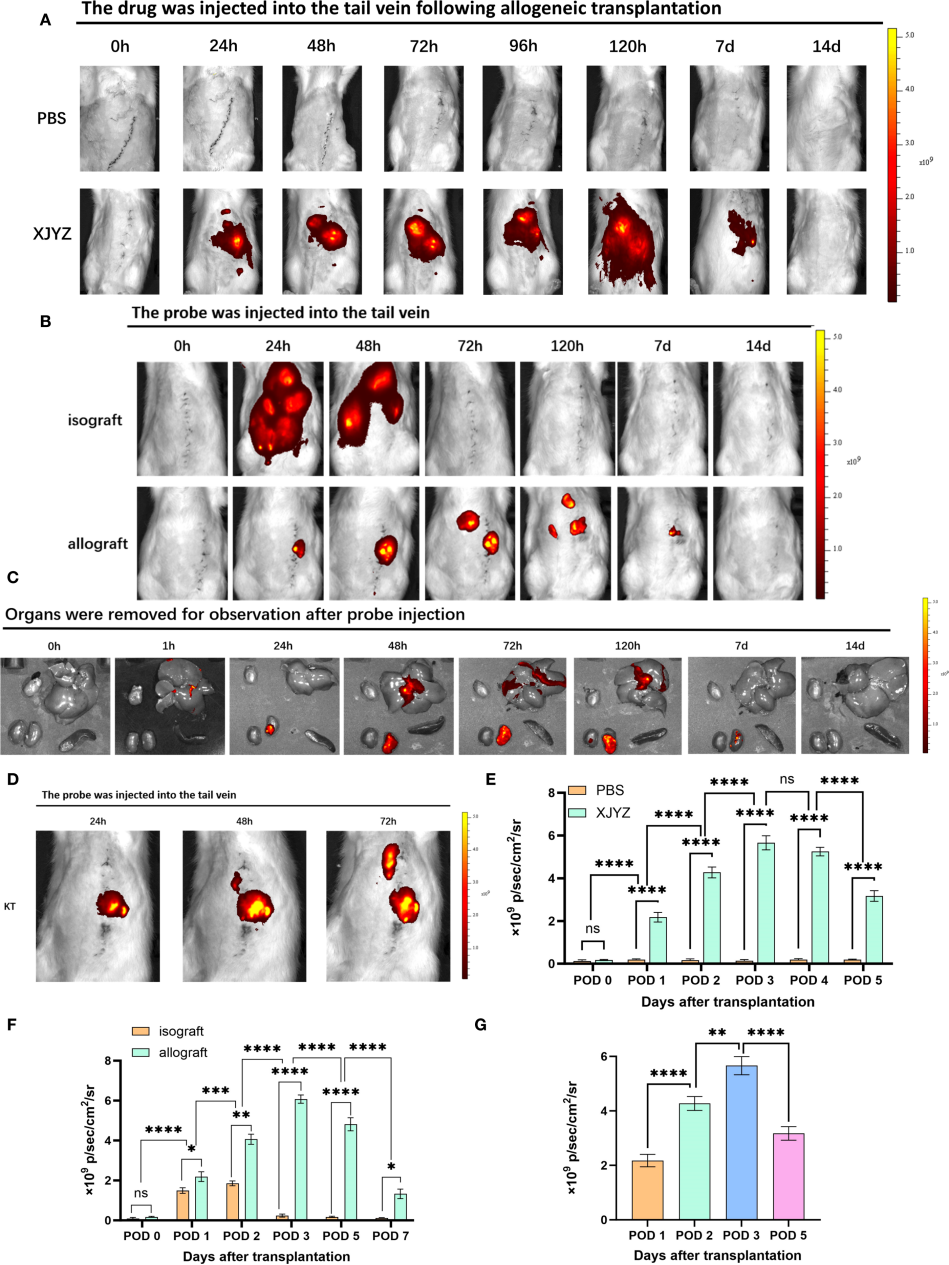
*In vivo* imaging of probe XJYZ for renal transplant rejection. **(A)** Metabolic profile of probe XJYZ *in vivo*. **(B)** Imaging for rejection monitoring by probe XJYZ. **(C)** Monitoring of rejection status in key organs post-transplantation. **(D)** Early rejection monitoring by XJYZ in the post-transplantation period. **(E)** Quantification of fluorescence intensity in the transplanted kidney during *in vivo* metabolism of probe XJYZ. **(F)** Quantitative analysis of fluorescence intensity in the transplanted kidney during rejection monitoring. **(G)** Quantitative fluorescence analysis of the transplanted kidney after harvest. Data in **(E–G)** are presented as mean ± standard deviation; ns denotes no statistically significant difference. *:P<0.05, **:P<0.01, ***:P<0.001, ****:P<0.0001.

To further validate whether probe XJYZ can achieve early detection of TCMR by targeting M1 macrophages in a rat renal transplantation model.

we first evaluated the metabolic profile of XJYZ in allogeneic rat renal transplants. Both the experimental and control groups utilized rats with allogeneic renal transplantation, wherein the experimental group received a 1 mM injection of XJYZ via the caudal vein of the recipients, while the control group was administered an equivalent volume of PBS, followed by imaging at designated time points. The control group showed baseline fluorescence signals (**Figure 7A**). In the experimental group, fluorescence primarily accumulated in the transplanted kidney region, with the signal demonstrating an increasing trend at 24, 48, and 72 hours post-injection, reaching its peak intensity at 72 hours. Due to the sustained presence of M1 macrophages within the transplanted kidney, the majority of the probe continued to be imaged at the graft site beyond 72 hours, while a minor portion was metabolized by the liver and the contralateral kidney. The fluorescent intensity in the transplanted kidney began to decline after the 7th day as the population of M1 macrophages decreased, returning to baseline levels by the 14th day (**Figure 7A**). Quantification of fluorescence signals in the transplanted kidney region (**Figure 7E**) showed that XJYZ accumulation was higher than controls from 1 to 5 days post-transplantation in allogeneic models.

To investigate the specificity of probe XJYZ for imaging TCMR in a rat kidney transplantation model, we established an allogeneic kidney transplantation model in Wistar-SD rats, with syngeneic SD-SD kidney transplantation serving as a control. The syngeneic transplantation group, owing to the close genetic relatedness of the rats, exhibited only a local inflammatory response postoperatively attributable to ischemia-reperfusion injury and surgical trauma, without manifesting significant rejection; in contrast, the allogeneic transplantation group demonstrated evident acute rejection.

Notably, we retained the contralateral native kidneys in the recipients to validate the specificity of probe XJYZ for rejection. To eliminate the interference of endogenous fluorescence, we performed *in vivo* fluorescence imaging in SD rats prior to probe injection (**Supplementary Figure 19**). The results showed that the autofluorescence signals from the abdominal organs were very weak and did not interfere with the probe’s fluorescence. Furthermore, given the inevitable ischemia-reperfusion injury in organ transplantation, which recruits inflammatory cells to the grafted kidney, a comparison was made between the early fluorescent imaging in ischemia-reperfusion injury and allogeneic transplantation models to mitigate nonspecific imaging (**Supplementary Figure 20**). In the allogeneic transplantation group, sustained activation of M1 macrophages maintained high fluorescence intensity imaging within 72 hours. Although M1 macrophages were recruited to the left kidney due to the inflammatory response in the ischemia-reperfusion group, resulting in initial imaging, their phenotype rapidly shifted and the probe was subsequently metabolized, leading to complete disappearance of fluorescence *in vivo* by 72 hours. This indicates that the high-intensity imaging from post-transplant rejection can distinguish nonspecific imaging caused by ischemia-reperfusion injury.

Following renal transplantation, recipients in both the allogeneic transplantation group (experimental group) and the syngeneic transplantation group (control group) received intravenous administration of XJYZ (1 mM) via the caudal vein and underwent imaging at designated time points. In the control group, rats with syngeneic kidney transplantation exhibited widespread fluorescence signals in the body at 24 hours post-injection. Due to the presence of only ischemia-reperfusion injury and surgical trauma in the control group, the recruited M1 macrophages in the transplanted kidney underwent a rapid phenotypic shift, and the probe was consequently metabolized swiftly *in vivo*, thus the fluorescent signal at 48 hours was predominantly localized to the liver and both kidneys. By 72 hours, the probe was completely metabolized, and the fluorescence signals in the recipients disappeared (**Figure 7B**).

In the allogeneic transplantation group, due to intense inflammatory and rejection responses, the probe XJYZ specifically accumulated in the transplanted kidney at 24 hours, whereas no fluorescent signal was observed in the contralateral native kidney. The fluorescence signals in the transplanted kidneys gradually increased over time, reaching a peak at 72 hours. To further activate the rejection response, M1 macrophages sustained their activity within the transplanted kidney, enabling the probe to persist *in vivo* and present a prolonged dynamic imaging process; until 120 hours, as the phenotypic shift of M1 macrophages occurred, the probe began to be gradually metabolized, and faint yet discernible fluorescent signals started to be observed in the liver and the contralateral native kidney. The transplanted kidneys continued to show significant fluorescence signals at 7 days, which completely disappeared by 14 days (**Figure 7B**).

We subsequently quantified the fluorescence signals in the transplanted kidneys (**Figure 7F**). At 48 hours post-transplantation, the fluorescence signals in the TCMR group were 2.18 times higher than those in the control group, indicating significant infiltration of M1 macrophages in allografts and a high risk of rejection. The period from 24 to 72 hours post-transplantation was identified as the most significant for M1 macrophage infiltration (**Figure 7D**). Imaging and quantitative analysis of fluorescence intensity in both syngeneic and allogeneic rat kidney transplants demonstrated that probe XJYZ enabled dynamic monitoring of early M1 macrophage infiltration. The specificity of XJYZ for monitoring rejection was sufficient to promptly reflect the occurrence of rejection.

To evaluate the impact of tissue penetration on fluorescence signals, we intravenously administered XJYZ via the tail vein post-transplantation and euthanized recipients at designated time points. Key organs (heart, liver, spleen, and kidney) were harvested for ex vivo imaging (**Figure 7C**). Quantitative analysis of the explanted renal grafts demonstrated peak fluorescence intensity at 72 hours post-transplantation, followed by gradual signal diminution (**Figure 7G**). This kinetic profile correlated with *in vivo* imaging findings, indicating that tissue penetrability does not compromise the detection efficacy of the XJYZ probe. Collectively, these results confirm that the XJYZ probe specifically accumulates in renal allografts during the early postoperative period, enabling noninvasive early-warning of TCMR in the rat allogeneic renal transplantation model.

### The probe XJYZ enables dynamic assessment of the therapeutic efficacy of TCMR treatment

**Figure 8:**
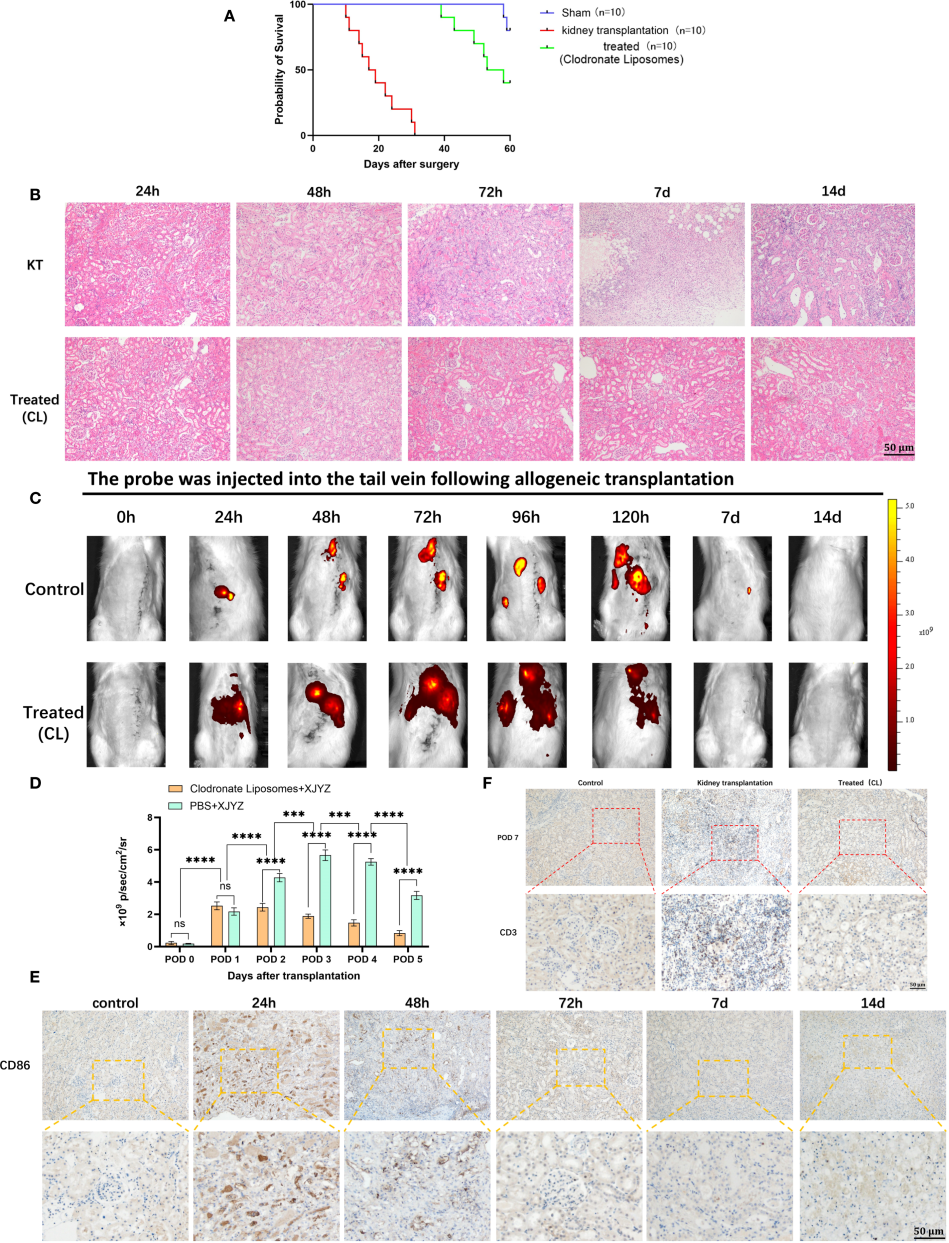
Dynamic assessment of early intervention efficacy by probe XJYZ. **(A)** Survival status of recipients following kidney transplantation in the clodronate liposome intervention group compared with the control group (same dose of PBS). **(B)** Hematoxylin and eosin (H&E) staining of transplanted kidneys in the intervention group and the control group. **(C)** Dynamic assessment of therapeutic efficacy for T-cell-mediated rejection (TCMR) using probe XJYZ. **(D)** Quantitative analysis of fluorescence intensity in the transplanted kidney region. **(E)** Infiltration of M1 macrophages in the transplanted kidneys before and after intervention. **(F)** T-cell infiltration in the transplanted kidneys at 7 days post-intervention. ns denotes no statistically significant difference. ***:P<0.001, ****:P<0.0001.

To further investigate whether probe XJYZ can dynamically assess treatment efficacy after rejection therapy, intervention was initiated at 24 hours post-transplantation in Wister-SD allogeneic renal transplant rats. The allograft treatment (intervention) group received clodronate liposomes (5 mg/kg) via tail vein injection at 24 hours and 72 hours post-transplantation - a drug known to specifically deplete macrophages *in vivo* (16). The allograft control group received an equal volume of PBS via tail vein at the same time points (**Supplementary Figure 22**).Notably, clodronate liposomes induced specific macrophage depletion, leading to a significant extension of recipient survival time to over 40 days, with half of the rats surviving beyond 60 days (**Figure 8A**).

Histopathological examination and XJYZ probe fluorescence imaging were performed in renal allograft recipients post-intervention. H&E staining revealed extensive inflammatory cell infiltration in control grafts at 72 hours, indicative of TCMR onset. Clodronate Liposomes intervention markedly mitigated interstitial inflammation and tubular injury in the treatment group (**Figure 8B**). *In vivo* fluorescence imaging demonstrated significantly attenuated signal intensity in renal allografts at 48 hours. While discernible fluorescence persisted in control grafts at day 7, complete signal resolution was observed in the intervention group by the 7-day endpoint (**Figure 8C**). Quantification of fluorescence intensity in the transplanted kidney showed that the control group had 2.2-fold higher intensity than the experimental group (**Figure 8D**). Additionally, fluorescence imaging results in the intervention group were consistent with immunohistochemical staining for anti-CD86 antibody: M1 macrophages gradually decreased after 24 hours of intervention and were successfully maintained at lower levels by 72 hours (**Figure 8E**).

To validate the effect of early intervention on rejection, recipients in both intervention and control groups were euthanized at day 7, and transplanted kidney tissues were subjected to immunohistochemical staining for anti-CD3 antibody. Results showed extensive T cell infiltration in the interstitium of control group grafts at day 7, whereas T cell counts in the intervention group approximated those of normal kidneys (**Figure 8F**). These findings demonstrate that early monitoring and intervention effectively alleviate rejection, reduce graft injury, and ultimately improve renal allograft outcomes.

Consequently, imaging with the XJYZ probe enables early risk stratification for graft rejection, provides real-time longitudinal monitoring of therapeutic response *in vivo*, and may serve as a complementary modality to conventional clinical surveillance. This approach facilitates preemptive intervention and promotes precision-based administration of immunosuppressive agents.

## Discussion

In summary, we developed the NIR fluorescent probe XJYZ, capable of enabling *in vivo* imaging specifically targeting M1 macrophages following renal transplantation, thereby facilitating early non-invasive diagnosis of transplant rejection. Our findings demonstrated that M1 macrophage infiltration occurred early within the allograft in a rat model of allogeneic renal transplantation, with the degree of infiltration quantitatively correlating directly with the severity of rejection. Macrophages upregulate glucose transporter 1 (GLUT1) during their polarization towards the pro-inflammatory phenotype. Leveraging this mechanism and the characteristic phagocytosis of the CY5 fluorophore by macrophages, we engineered probe XJYZ to target M1 macrophages for fluorescence imaging. *In vitro* studies confirmed the probe’s specificity for imaging M1 macrophages without labeling undifferentiated M0 or anti-inflammatory M2 macrophages. *In vivo* experiments further established a positive correlation between the NIRF signal intensity of XJYZ and both the extent of M1 macrophage infiltration and the severity of TCMR. Furthermore, early post-transplant targeted intervention specifically modulating macrophage activity significantly ameliorated adverse renal allograft outcomes. This suggests that XJYZ holds potential utility in the development of targeted therapeutic agents.

TCMR is a common type of rejection reaction after kidney transplantation, and early monitoring and early warning of TCMR are crucial for improving the long-term prognosis of the transplanted kidney and enhancing patient survival rates (17). Although renal biopsy remains the gold standard for diagnosing TCMR, its invasive nature, high cost, and delayed results are still problematic. Therefore, how to non-invasively and predict TCMR in advance through other means is a problem that needs to be solved. In this study, we successfully demonstrated that monitoring M1 macrophages via probe XJYZ enables early prediction of rejection. Compared with previous studies monitoring immune cells (e.g., T cells, granzyme B) and damage markers (8, 18), XJYZ can detect initial rejection events (M1 macrophage infiltration) days before the onset of histological damage or functional decline. It allows non-invasive real-time visualization of M1 macrophage recruitment, advancing the rejection warning window by several days for early monitoring. Furthermore, the current clinical diagnosis of acute rejection post-transplantation primarily relies on biopsy or elevated serum creatinine levels, both of which are lagging indicators of established injury (16). Patients often present with symptoms like fever and anuria at this stage (19), requiring high-dose immunosuppressive pulse therapy to ameliorate symptoms. However, excessive immunosuppression leads to severe side effects, including neoplasms and infections (20) (21). Thus, early monitoring and intervention are critical for avoiding overtreatment with immunosuppressants, minimizing side effects, and improving outcomes.

Although T lymphocytes constitute the primary cellular mediators of T cell-mediated rejection (TCMR), both published literature and our transcriptomic analyses indicate that macrophages play a pivotal role in TCMR pathogenesis. This is substantiated by a significant correlation between M1 macrophage infiltration and T cell activity, as evidenced through immunohistochemical co-localization of CD86^+^ and CD3^+^ cells within renal allograft sections. Prior investigations have established that during early post-transplant phases, macrophages function as antigen-presenting cells (APCs), processing and presenting donor-derived antigenic peptides via MHC class II molecules to CD4^+^ T cell receptors (22). Concurrently, costimulatory molecules expressed on macrophage surfaces—specifically B7-1 (CD80) and B7-2 (CD86)—engage CD28 on T lymphocytes, delivering the critical second signal essential for T cell activation and clonal expansion (23). In the meantime, IL-12 secreted by M1 macrophages drives CD4^+^ T cell differentiation into Th1 cells, promoting cellular immune rejection (24). Secretion of TNF-α and IL-1β directly activates T cells, enhancing inflammatory responses (25). Recruitment of M1 macrophages facilitates T cell infiltration and functional activation, while cytokines secreted by T cells further promote macrophage polarization toward the M1 subtype, forming a positive feedback loop that amplifies inflammatory reactions (26).Additionally, our sequencing results revealed that early infiltration of M1 macrophages is associated with dendritic cells (DCs) and natural killer (NK) cells. We postulate that M1 macrophages, in conjunction with DCs and NK cells, collectively drive inflammation and induce tissue injury during the early post-transplantation period, which is consistent with previously reported findings (27).

Energy metabolism plays a crucial regulatory role in the polarization of macrophages (28). M1 macrophages primarily rely on glycolysis for energy metabolism, whereas M2 macrophages mainly derive their energy from fatty acid oxidation (29). GLUT1 (Glucose Transporter 1) plays a primary role in glycolysis. Studies on tumors and inflammatory diseases have shown that in a pro-inflammatory environment, macrophages upregulate GLUT1 expression, thereby enhancing glycolytic capacity to meet their high energy metabolic demands and polarizing toward the M1 subtype (30). In the field of oncology, molecular imaging targeting GLUT1 has been successfully employed to visualize M1 macrophage distribution, thereby delineating tumor boundaries as reported in the literature (31). In the field of transplantation, there has been no report on monitoring rejection through GULT1. Our study focused on the expression of GLUT1 on M1 macrophages after transplantation. Sequencing of early post-transplantation specimens demonstrated that when M1 macrophages exert pro-inflammatory functions in the early stage, GLUT1 expression is upregulated accordingly. Based on this property, we successfully capitalized on GLUT1 to achieve targeted therapy for M1 macrophages, while the CY5 fluorophore within the probe structure was also instrumental in the targeting process. Monitoring GLUT1 expression can effectively reflect the status of inflammation and rejection. Although GLUT1 is widely expressed in tissues and organs such as the brain, nervous system, muscles, and red blood cells *in vivo* (32, 33), targeting GLUT1 may potentially lead to systemic nonspecific imaging. However, the presence of hydrophobic groups such as multiple aromatic rings and a long carbon chain in the CY5 fluorophore causes probe XJYZ to be readily phagocytosed by macrophages (34), thereby preventing systemic nonspecific imaging; furthermore, this design leverages the inherent property of macrophages to phagocytose lipidic substances (35), thus also avoiding nonspecific imaging that could arise from upregulation of GLUT1 following the activation of transplanted immune cells such as T cells and B cells.

*In vivo* imaging findings with probe XJYZ demonstrated precise concordance with histopathological assessment via hematoxylin and eosin (H&E) staining of renal allografts, consistent with our predictions. At the 72-hour post-transplantation timepoint, allograft sections revealed extensive interstitial inflammatory cell infiltration accompanied by acute tubulitis—cardinal histopathological features diagnostic of T cell-mediated rejection (TCMR)—which corresponded temporally with peak near-infrared fluorescence (NIRF) signal intensity *in vivo*. Moreover, progressively escalating NIRF signals detected at 24-hour and 48-hour intervals served as early warning indicators of impending TCMR development. By postoperative day 14, complete signal resolution was observed alongside H&E evidence of extensive medullary fibrosis. This transition aligns with established literature documenting macrophage polarization toward the pro-fibrotic M2 phenotype during fibrotic phases (36), suggesting signal attenuation may be attributable to diminished M1 subpopulation density and concurrent expansion of repair-promoting M2 macrophages facilitating tissue repair.

Compared with traditional biomarkers, XJYZ can capture subtle inflammatory signals at an early stage, thereby enhancing the sensitivity of monitoring. XJYZ helps offer new insights into clinical rejection monitoring by identifying patients with early and active M1 macrophage infiltration, enabling the detection of high-risk individuals and timely targeted intensification of therapy to potentially prevent the occurrence of full-blown rejection. Conversely, patients with weak and stable imaging signals can be considered safe candidates for reducing immunosuppressive intensity, which helps minimize long-term drug toxic side effects (such as infections, malignancies, and metabolic diseases). The probe XJYZ can complement current clinical diagnostic methods for rejection, allowing planned biopsy for definitive histological diagnosis once significant fluorescence intensity appears. Additionally, the probe XJYZ can synergize with other biomarkers, such as donor-derived cell-free DNA (dd-cfDNA) (37), to collectively establish a comprehensive non-invasive rejection detection system.

Nevertheless, several inherent limitations of XJYZ warrant consideration. The penetration depth of NIR-I fluorescence imaging imposes fundamental constraints on visualizing deep anatomical structures such as renal allografts (38)—a limitation circumvented in our experimental design through ex vivo imaging of explanted specimens, which confirmed concordance between *in situ* and post-explantation signal distribution. Future investigations should prioritize developing dual-modality probes integrating fluorescence with positron emission tomography (PET) to enable deep-tissue interrogation, thereby enhancing clinical translatability. Advances in single-cell RNA sequencing have revealed unprecedented functional heterogeneity within macrophage populations (39, 40); while XJYZ targets the broad M1 macrophage classification, it currently lacks resolution to distinguish functionally divergent subsets within this spectrum due to biological complexity. Furthermore, clinical translation necessitates rigorous validation through comprehensive safety assessments, including detailed toxicological characterization and pharmacokinetic profiling. Addressing these limitations remains imperative for transitioning XJYZ from preclinical utility to clinical implementation, ultimately improving long-term renal allograft outcomes.

## Data Availability

The original contributions presented in the study are publicly available. This data can be found here: GSE303904. https://www.ncbi.nlm.nih.gov/geo/query/acc.cgi?acc=GSE303904.
