# Supplementary material for: Visualizing early allograft rejection: an M1 macrophage-specific GLUT1 probe predicts TCMR onset in renal transplantation
Source: Front Immunol. 2025 Oct 2;16:1670370. doi: 10.3389/fimmu.2025.1670370 (PMC12527843; doi:10.3389/fimmu.2025.1670370)
Supplement: Supplementary file 1 [file DataSheet1.pdf]

Visualizing Early Allograft Rejection: An M1 Macrophage-Specific GLUT1 Probe Predicts TCMR Onset in Renal Transplantation

Running Title: GLUT1-Targeted Molecular Probe for Monitoring Post-Transplant Rejection

Zhaoxiang Wang[1, #]; Fanchao Wei[1, #];Dayuan Huang[1, #]; Ruochen Qi[1]; Shichao Han[1]; Changhong Shi[2]; Hongtao Song[1]; Yuxuan Du[1]; Zhengxuan Li[1]; Lang LI[3]; Jingliang Zhang[1, *];Shuaijun Ma[1, *]; Weijun Qin[1, *];

[1,] Department of Urology, Xijing Hospital, Air Force Medical University, Xi'an, Shaanxi 710032, China

[2,] Division of Cancer Biology, Laboratory Animal Center, Air Force Medical University, Xi'an, Shaanxi 710032, China

[3,] Skills Training Center, Xijing Hospital, Air Force Medical University, Xi'an, Shaanxi 710032, China

Correspondence E-mail: qinwj@fmmu.edu.cn

Contents of this SI file include:

• Supplementary Figure 1 to 22.

• Supplementary Table 1 to 2.

• Supplementary Methods including information on library and XJYZ (Supplementary Figure 9 to 11, 21 to 22, Supplementary Scheme 1).

List of Supplementary Figures:

Supplementary Fig. 1. Schematic diagram of rat kidney transplantation surgery

Supplementary Fig. 2. Perfusion of transplanted kidney and vesicoureteral reimplantation

Supplementary Fig. 3. B-ultrasound observation of blood supply of transplanted kidney and bladder filling status

Supplementary Fig. 4. Masson staining of transplanted kidneys at different time points

Supplementary Fig. 5. Expression of M1 Macrophages in Renal Allograft Tissue

Supplementary Fig. 6. Interaction between M1 Macrophages and T Cells at 72 Hours Post-Transplantation

Supplementary Fig. 7. Expression Difference of GLUT1 Between M1 and M2 Macrophage

Supplementary Fig. 8. Expression of GLUT1 protein in transplanted kidneys

Supplementary Fig. 9. GLUT1 Expression in Renal Allograft Tissue

Supplementary Fig. 10. Composition of Fluorescent Moieties in the Library

Supplementary Fig. 11. Composition of Carbohydrate Scaffolds in the Library

Supplementary Fig. 12. Library Combinatorial Strategy

Supplementary Fig. 13. Schematic Diagram of Primary Bone Marrow Cell Isolation and Polarization

Supplementary Fig. 14. Schematic Diagram of Screening for Optimal Targeting Probe

Supplementary Fig. 15. Polarization Status of M1 and M2 Macrophages

Supplementary Fig. 16. In Vitro Safety Validation of the Probe

Supplementary Fig. 17. Cell apoptosis after probe co-incubation.

Supplementary Fig. 18. In Vivo Safety Validation of the Probe

Supplementary Table 1. In Vivo Safety Validation (Hematological Parameters)

Supplementary Fig. 19. Suppression of Rat Autofluorescence

Supplementary Fig. 20. Probe imaging in ischemia-reperfusion injury

Supplementary Fig. 21. Probe XJYZ in vivo imaging

Supplementary Fig. 22. Schematic Diagram of Early Post-Transplantation Intervention

Supplementary Fig. 23. Infiltration of M1 Macrophages in Grafts After Intervention

Supplementary Fig. 24. Quantification of Fluorescence Intensity in Graft Tissues After Intervention

Supplementary Fig. 25. Changes in M1 Macrophage Infiltration in Renal Allografts Between Intervention and Non-Intervention Groups

Supplementary Scheme 1. Synthetic Pathway of XJYZ

Supplementary Fig. 26.HR-MS spectrum of XJYZ

Supplementary Fig. 27.HPLC and mass chromatogram of XJYZ

Supplementary Table 2. Primer Sequences

Supplementary Table 3. Gene set associated with M1 macrophages

Supplementary Table 4. Western blot raw data

Supplementary Results

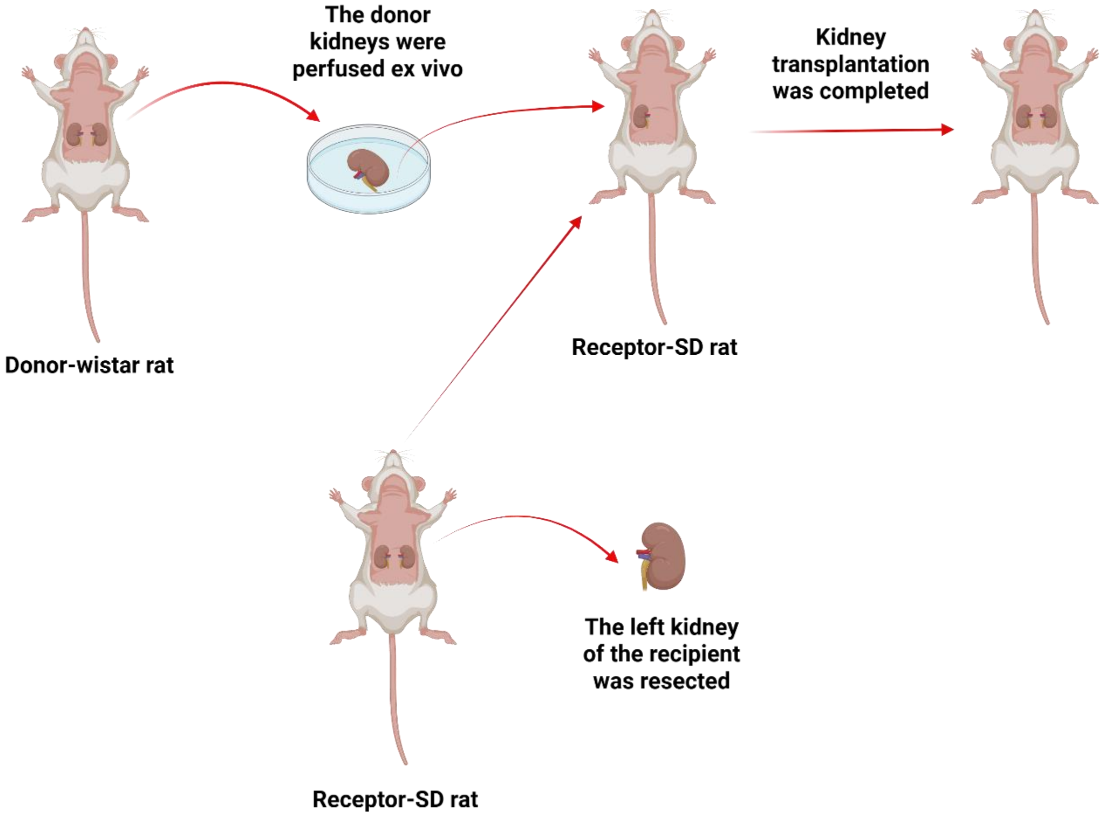

Supplementary Fig. 1 . Schematic diagram of rat kidney transplantation surgery

Wistar rats (150-300 g) were used as donors and SD rats as recipients to perform orthotopic left kidney transplantation. After removal of the donor kidney, it was perfused ex vivo and kept on ice with warm ischemia time controlled within 5min, followed by transplantation surgery. The renal artery was anastomosed end-to-end, the renal vein was anastomosed through an end-to-end cannula, and the ureter was reimplanted into the bladder.

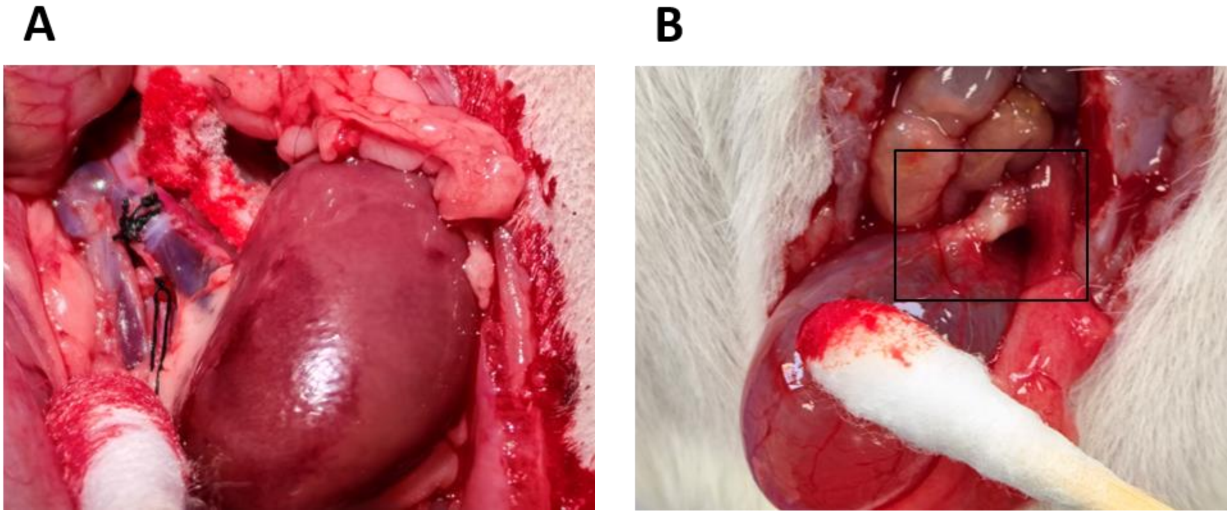

Supplementary Fig. 2. Perfusion of transplanted kidney and vesicoureteral reimplantation

Panel A shows the renal reperfusion after the renal arteriovenous anastomosis was completed, in which the renal artery was anastomosed end-to-end, and the renal vein was anastomosed by the "cannula method". Panel B illustrates uretero-bladder reimplantation after transplantation.

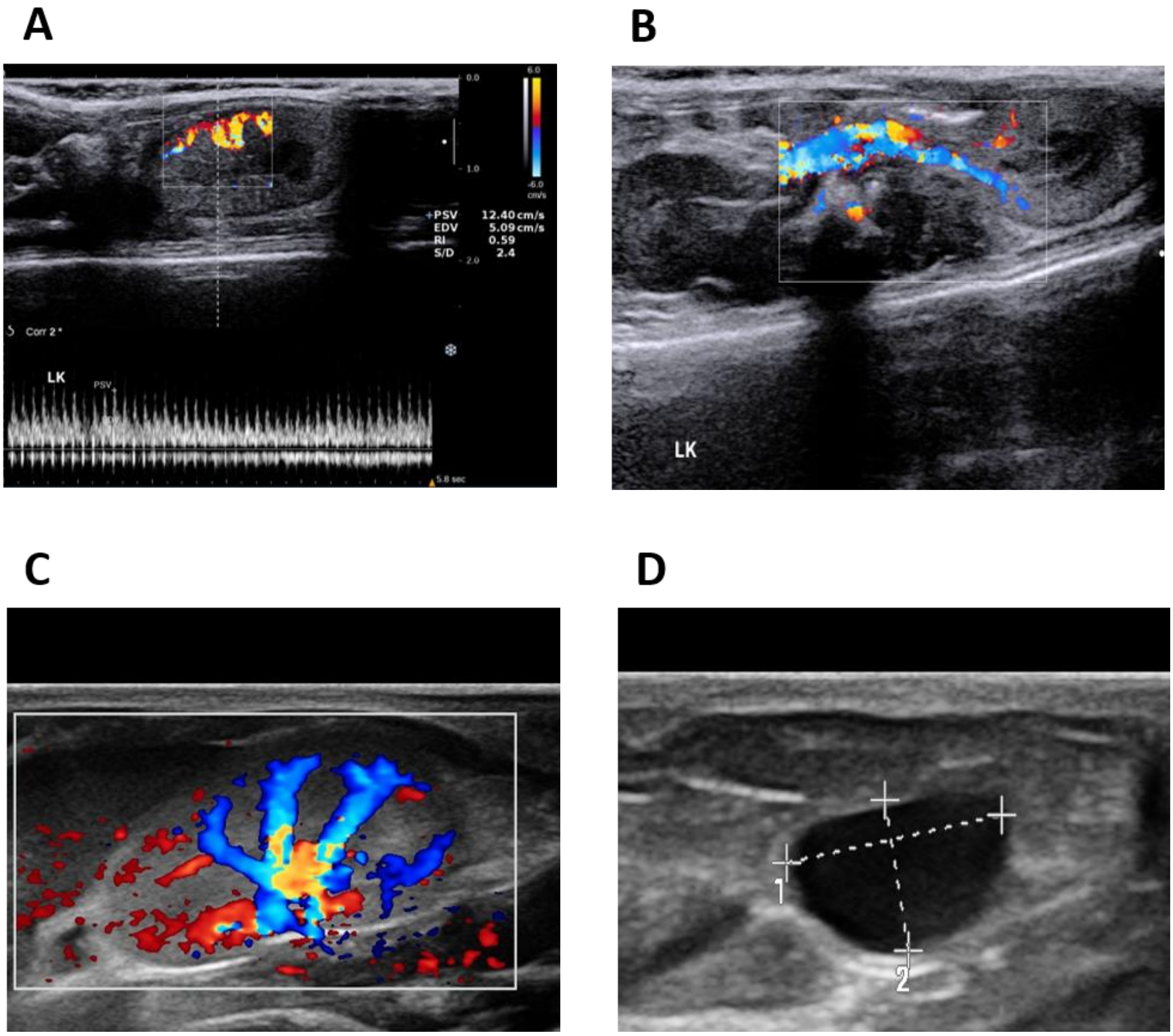

Supplementary Fig. 3. B-ultrasound observation of blood supply of transplanted kidney and bladder filling status

Panel A shows the results of B-ultrasound after operation to verify that the operation was successful, the blood flow of the transplanted kidney was recanalized, and the transplanted renal artery was perfused well. Panel B shows the blood flow at the renal hilum, and the blood flow of the transplanted renal artery and vein and the inferior vena cava of the abdominal aorta is unobstructed. Panel C demonstrated good venous return of the transplanted kidney without venous thrombosis or blood stasis. Panel D verified

that the effect of ureterovesical replantation was good, and the bladder showed a liquid dark area under B-ultrasound with normal filling status.

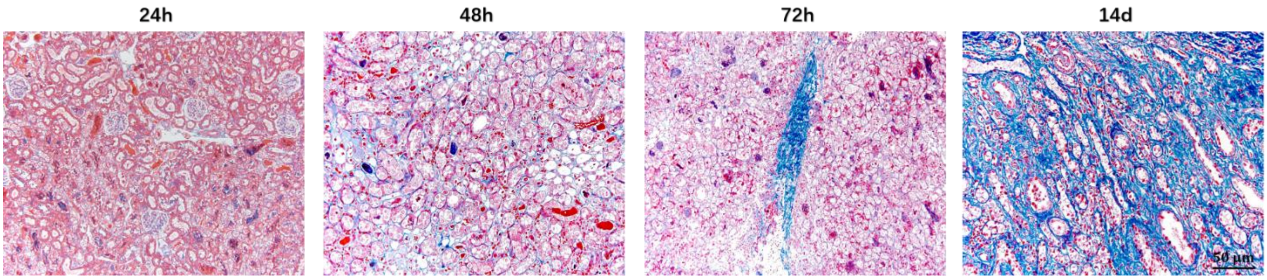

Supplementary Fig. 4. Masson staining of transplanted kidneys at different time points

Masson staining was performed on the transplanted kidney at different time points, and the fibrosis of glomeruli and renal tubules was observed in different degrees by Masson staining at 24 hours. It was considered that the injury of glomeruli and renal tubules caused by ischemia-reperfusion during transplantation was repaired. The degree of fibrosis in the renal allograft was mild at 48 hours, and the degree of fibrosis in the interstitium was gradually significant at 72 hours. At 14 days, there was obvious extensive fibrosis in the interstitium. Combined with HE staining, it was considered that a large number of M2 macrophages infiltrated in the interstitium, played a role in fibrosis repair, and the recruitment and continuous infiltration of T cells led to glomerular endothelial injury. Proinflammatory factors, such as TGF-β, activate fibroblasts and promote collagen deposition.

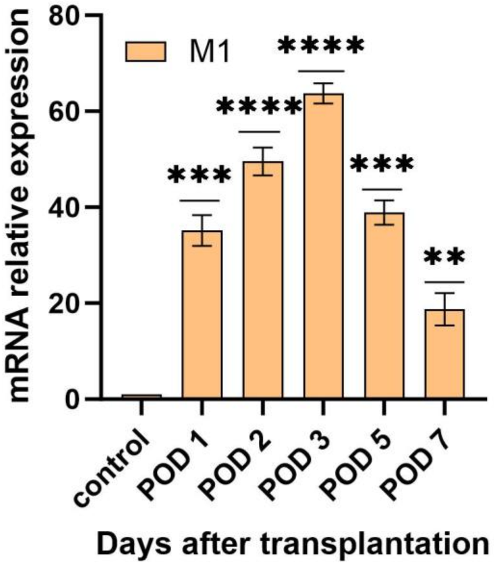

Supplementary Fig. 5. Expression of M1 Macrophages in Renal Allograft Tissue

Renal tissues were obtained at different time points after transplantation, and M1 macrophages were detected by qPCR to observe the infiltration of M1 macrophages in the transplanted kidney, which supplemented the CD86 immunohistochemistry results at different time points after transplantation. Primer designs are shown in Supplementary Table 2. All bars show SD ± mean (n=5).

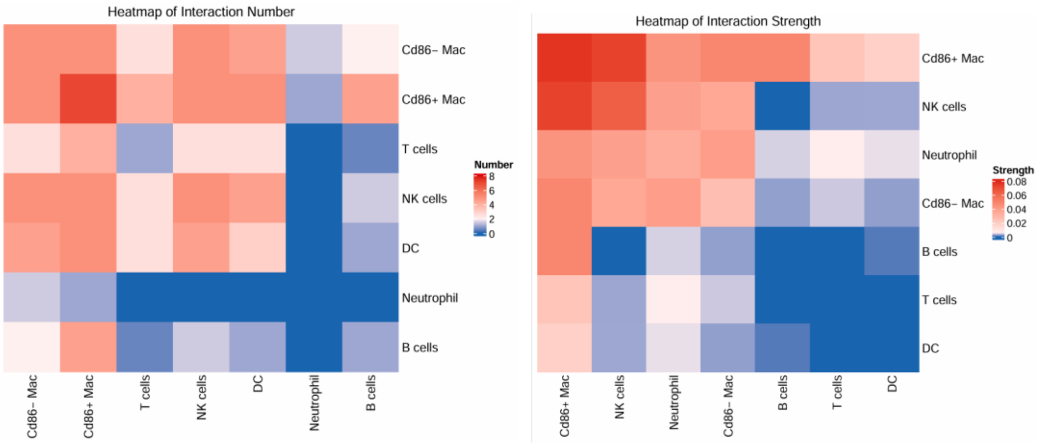

Supplementary Fig. 6. Interaction between M1 Macrophages and T Cells at 72 Hours Post-Transplantation

At 72 hours after transplantation, single cell suspension was prepared from renal allograft samples for single cell sequencing. The results showed that the number and interaction ability of T cells were highly correlated with M1 macrophages.

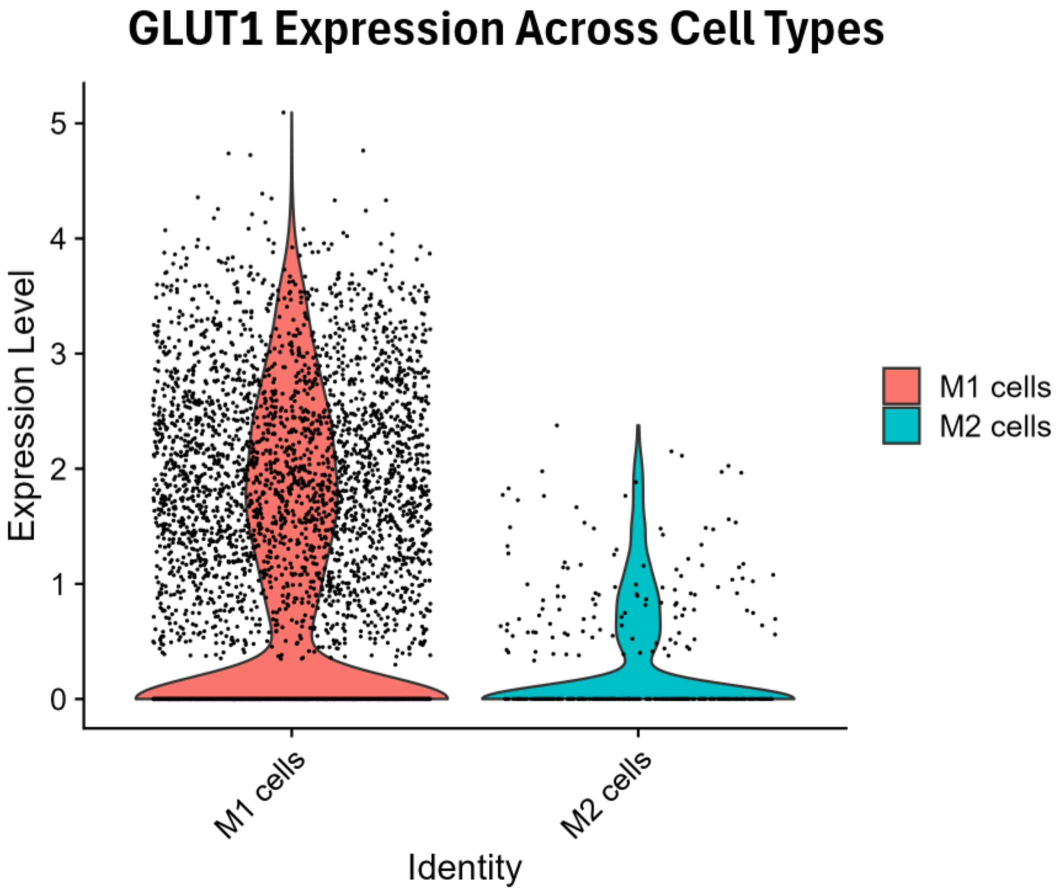

Supplementary Fig. 7. Expression Difference of GLUT1 Between M1 and M2 Macrophages

At 72 hours after transplantation, the number of M1 macrophages was significantly higher than that of M2 macrophages, and the expression of GLUT1 was highly correlated with that of M1 macrophages.

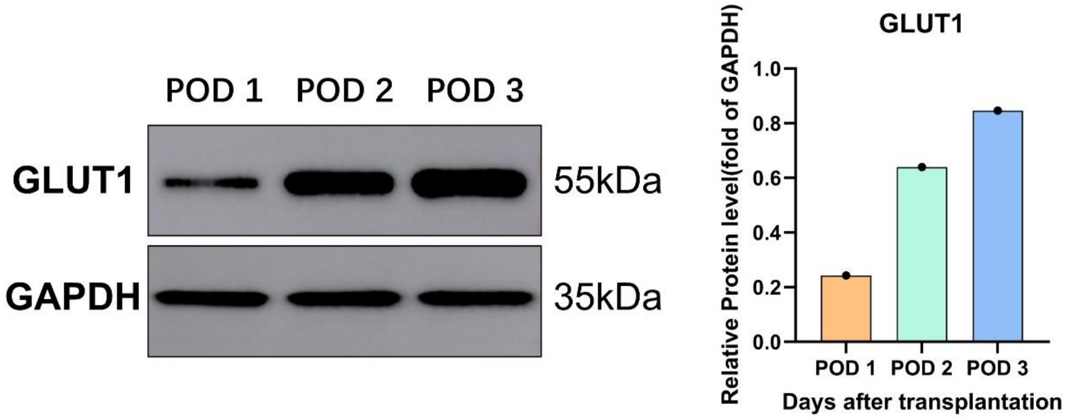

Supplementary Fig. 8. Expression of GLUT1 protein in transplanted kidneys

Western blot analysis of GLUT1 protein expression in transplanted kidneys indicated that GLUT1 expression increased in a time-dependent manner within the initial 72-hour period, with the raw data from the Western blot assays provided in Supplementary Table 4.

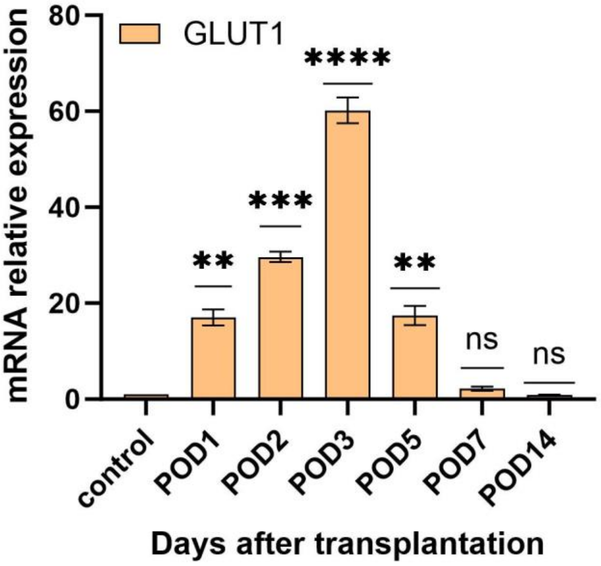

Supplementary Fig. 9. GLUT1 Expression in Renal Allograft Tissue

The renal tissues were obtained at different time points after transplantation, and the expression of GLUT1 in the transplanted kidney was detected by qPCR. The GLUT1 expression in the transplanted kidney was observed. Primer designs are shown in Supplementary Table 2. All bars show SD ± mean (n=5).

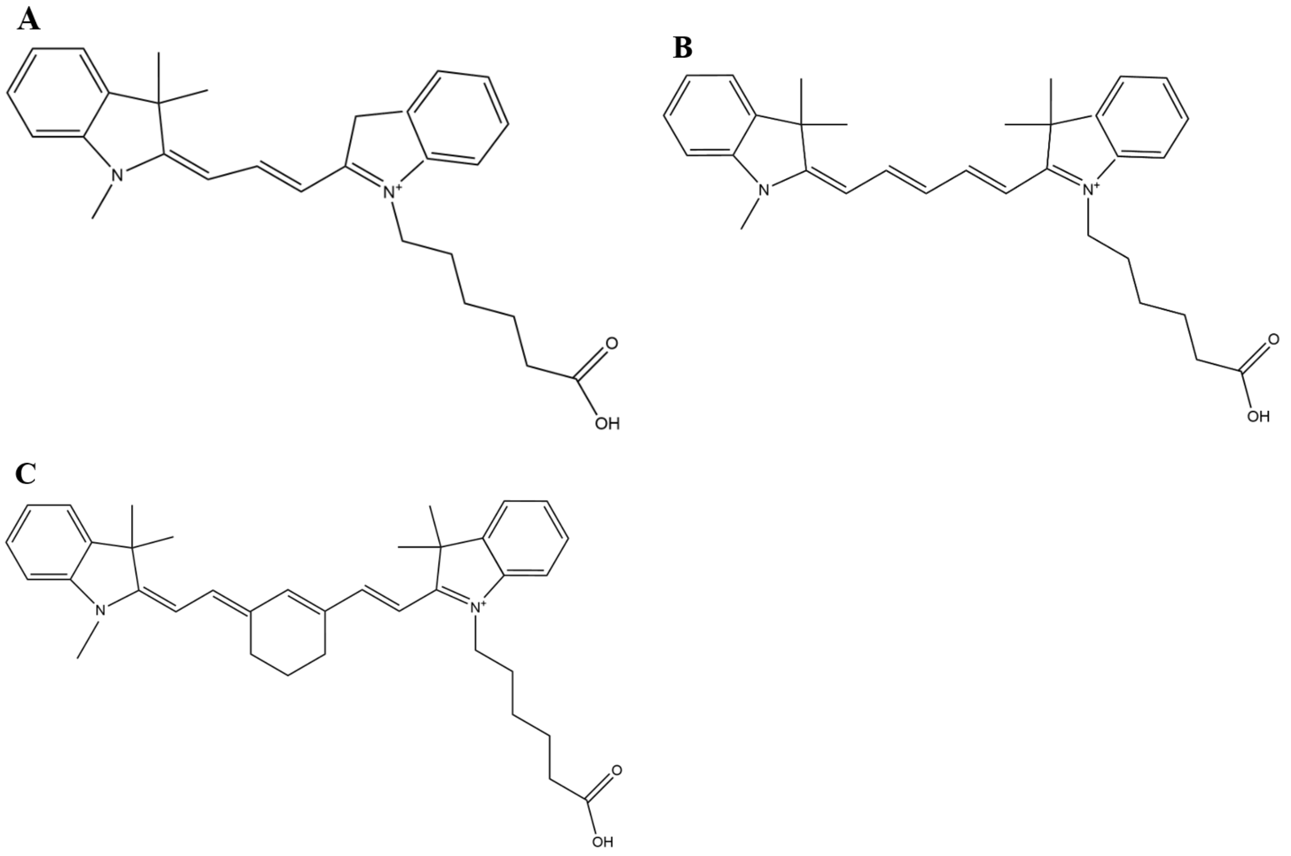

Supplementary Fig. 10. Composition of Fluorescent Moieties in the Library

1

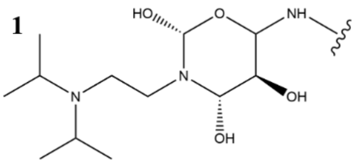

2

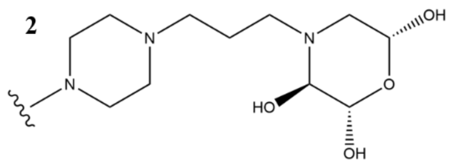

3

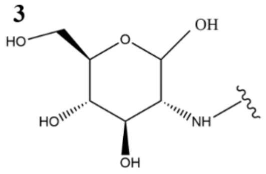

4

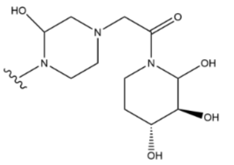

5

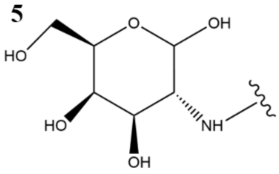

6

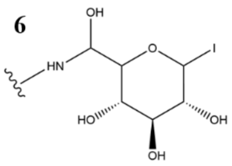

7

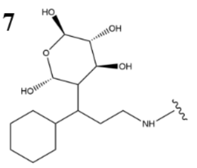

8

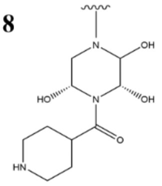

9

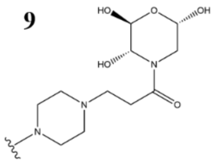

10

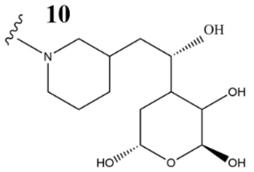

Supplementary Fig. 11. Composition of Carbohydrate Scaffolds in the Library

	1 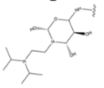	2 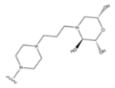	3 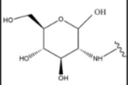	4 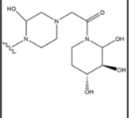	5 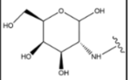	6 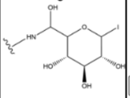	7 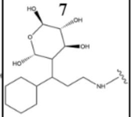	8 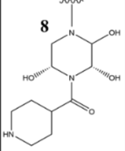	9 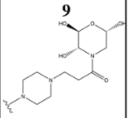	10 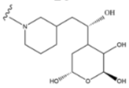
A 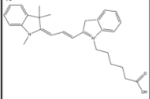	A-1	A-2	A-3	A-4	A-5	A-6	A-7	A-8	A-9	A-10
B 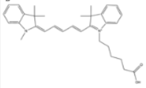	B-1	B-2	B-3	B-4	B-5	B-6	B-7	B-8	B-9	B-10
C 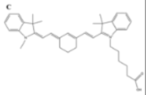	C-1	C-2	C-3	C-4	C-5	C-6	C-7	C-8	C-9	C-10

Supplementary Fig. 12. Library Combinatorial Strategy

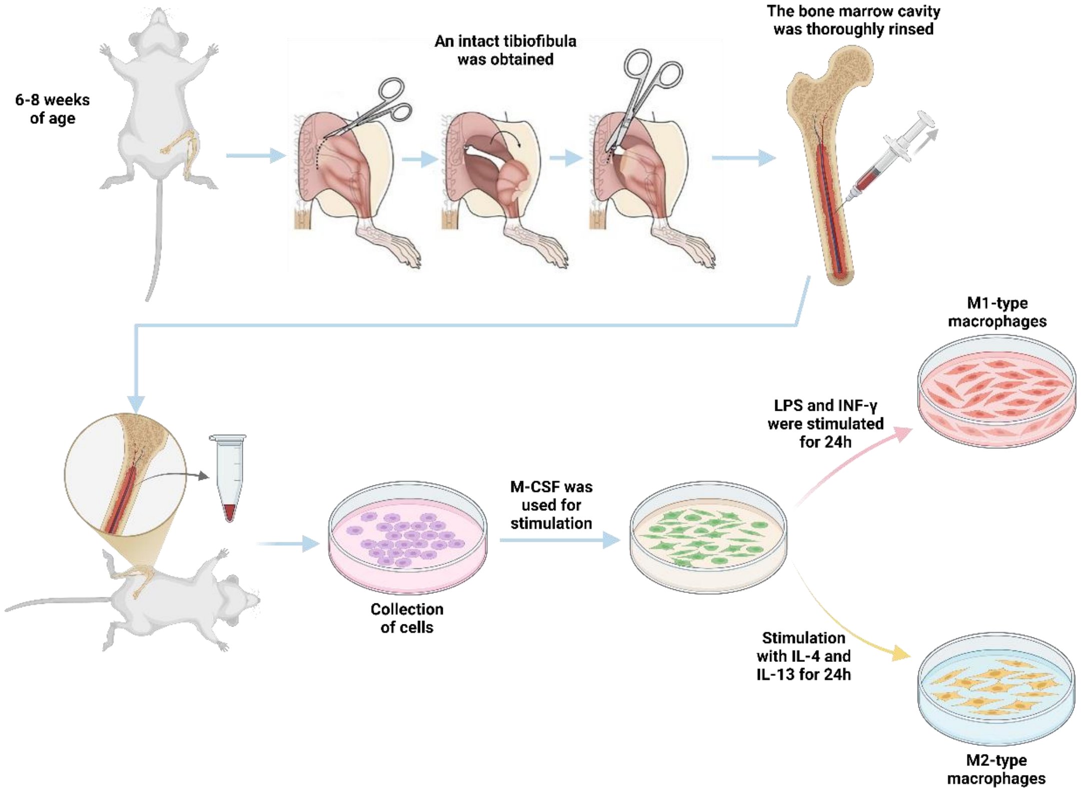

Supplementary Fig. 13. Schematic Diagram of Primary Bone Marrow Cell Isolation and Polarization

Six to eight weeks old mice were sacrificed and the leg skin was cut, the thigh root muscles were removed, and the entire leg was cut along the hip joint. The tibia and perifemoral muscles were removed with scissors to obtain intact femur and tibia. The epiphysis at both ends of the femur and tibia was excised, and the bone marrow was entered from the end with a 23G needle, which was rinsed out and collected. BMDM were generated by stimulation with M-CSF and subsequently stimulated to M1 macrophages with LPS and IFN-γ, and M2 macrophages were generated by stimulation with IL-4 and IL-13.

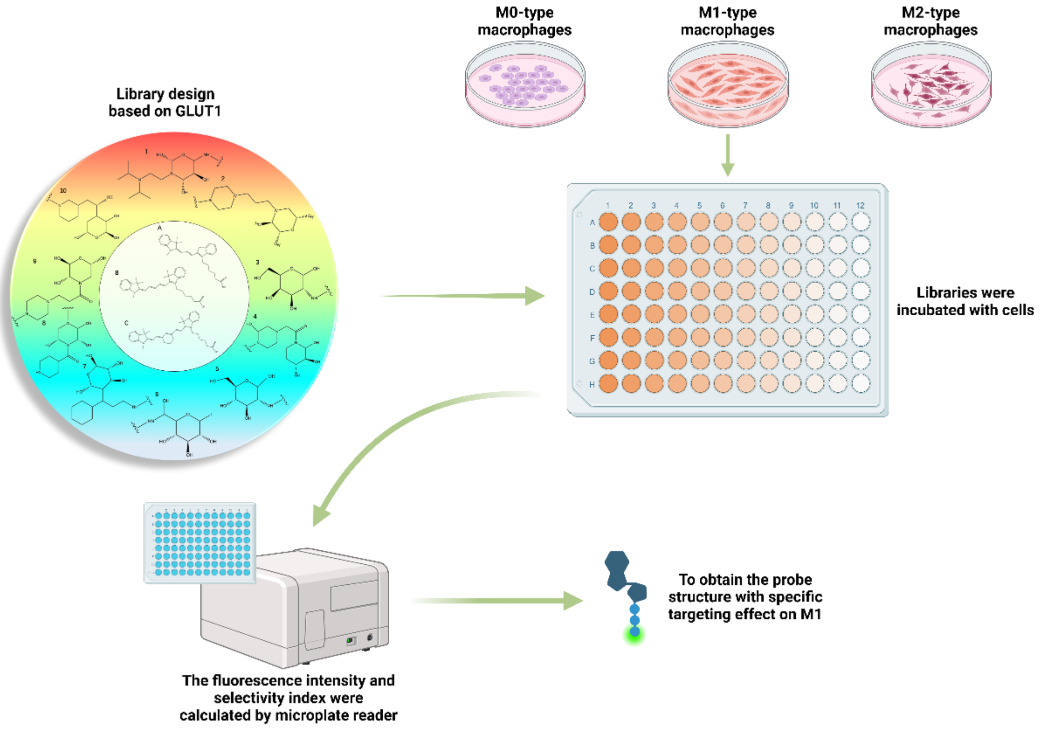

Supplementary Fig. 14. Schematic Diagram of Screening for Optimal Targeting Probe

The cultured M0, M1 and M2 macrophages were spread in 96-well plates, and the compounds in the library were co-incubated with the cells, respectively. After washing the compounds, the fluorescence intensity was detected by microplate reader to obtain the probe structure with the strongest selectivity for M1 macrophages.

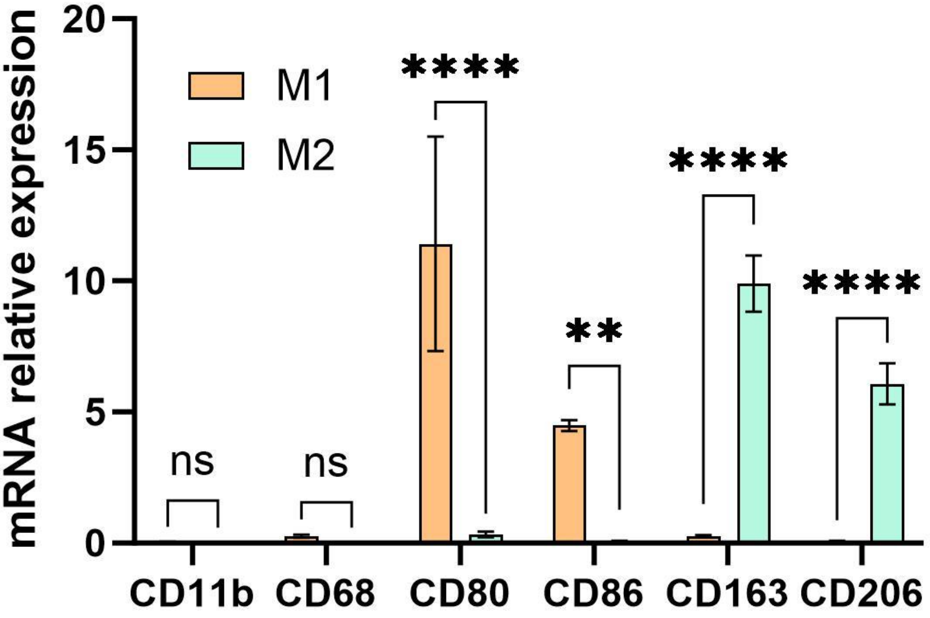

Supplementary Fig. 15. Polarization Status of M1 and M2 Macrophages

qPCR was used to detect M1 macrophage markers (CD80 and CD86) and M2 macrophage markers (CD163 and CD206) to verify cell polarization. Primer designs are shown in Supplementary Table 2. All bars show SD ± mean (n=5).

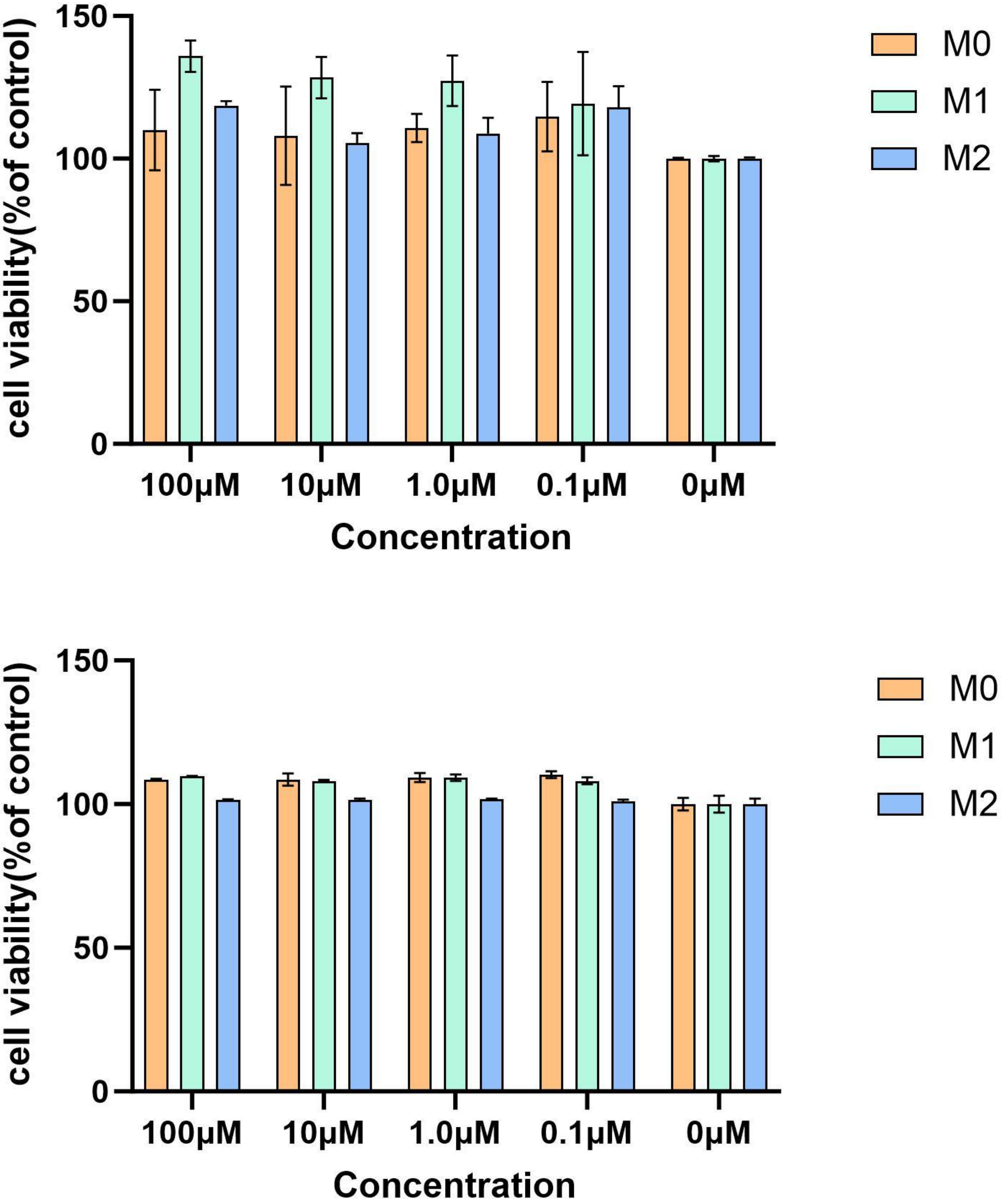

Supplementary Fig. 16. In Vitro Safety Validation of the Probe

CCK8 assay was used to verify the effect of the probes on cell survival in vitro. Four sets of concentration gradients were set, and the results proved that the probe had no effect on cell survival. All bars show SD ± mean (n=5).

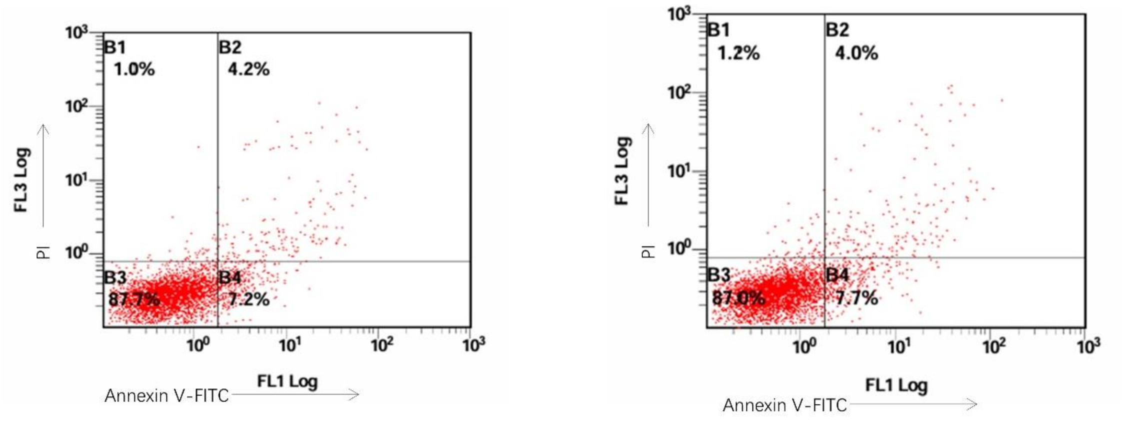

Supplementary Fig. 17. Cell apoptosis after probe co-incubation.

The experiment was repeated twice to enhance experimental accuracy. After co-incubating XJYZ with M1-type macrophages, Annexin V-FITC/PI double staining technique was used, and the results from the two repeated experiments showed that apoptotic cells accounted for 11.4% and 11.7% of the total cells, respectively. This indicates that the cytotoxicity of probe XJYZ is at an acceptable level.

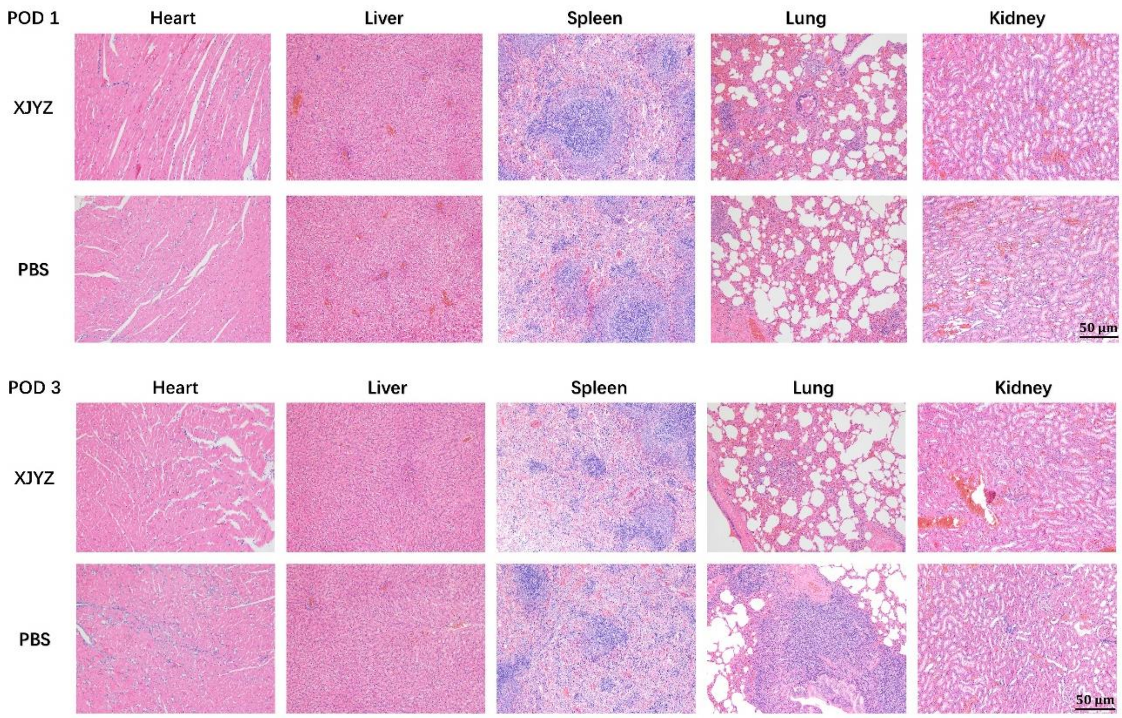

Supplementary Fig. 18. In Vivo Safety Validation of the Probe

The experimental group of healthy SD rats were injected with 1 mM probe XJYZ via the tail vein, while the control group was injected with the same dose of PBS. The rats were sacrificed at 1 day and 3 days post-injection, respectively. Key organs (heart, liver, spleen, lungs, and kidneys) were harvested for pathological analysis. The results demonstrated that the probe did not cause damage to the major organs in vivo.

Supplementary Table 1. In Vivo Safety Validation (Hematological Parameters)

Hematological parameters in whole blood of healthy SD rats following administration of probe XJYZ or PBS for 24h.(n=3)

	XJYZ	PBS	Unit	Reference Range
WBC	7.75 ± 1.02	4.17 ± 1.53	10^{-9}/L	3.50-9.50
NEUT#	2.03 ± 1.34	3.57 ± 1.16	10^{-9}/L	1.80-6.30
NEUT%	0.47± 0.13	0.52± 0.18		0.40-0.75
Lymph#	5.68 ± 0.65	4.17 ± 1.54	10^{-9}/L	1.10-5.70
Lymph%	0.73 ± 0.17	0.80 ± 0.03		0.50-0.90
Mono#	0.54 ± 0.07	0.25 ± 0.09	10^{-9}/L	0.10-0.60
Mono%	0.07 ± 0.02	0.05 ± 0.07		0.03-0.10
RBC	9.55 ± 0.34	7.81 ± 0.37	10^{-12}/L	6.00-10.00
HGB	145.0 ± 7.36	142.0 ± 10.14	g/L	110-145
HCT	42.4 ± 2.98	41.05 ± 1.54		30.6-45.1
MCV	50.7 ± 3.41	47.1 ± 3.86	fL	48.2-58.3
MCH	17.3 ± 0.18	15.2 ± 1.27	pg	13.8-19
MCHC	341 ± 1.45	327 ± 3.61	g/L	326-354
RDW%	0.12 ± 0.01	0.05 ± 0.07		0.04-0.15
RDW	38.4 ± 5.68	50.01 ± 3.71	fL	37-54
PLT	325 ± 10	273 ± 24	10^{-9}/L	125-350
MPV	7.10 ± 1.34	6.80 ± 2.39	fL	4.0-12.0
PDW	14.7 ± 0.32	15.4 ± 0.93	fL	12.0-18.0
PCT	0.53 ± 0.07	0.47 ± 0.24		

Hematological parameters in whole blood of healthy SD rats following administration of probe XJYZ or PBS for 3d.(n=3)

	XJYZ	PBS	Unit	Reference Range
WBC	6.43 ± 1.17	3.98 ± 2.19	10^{-9}/L	3.50-9.50
NEUT#	3.78 ± 2.36	3.14 ± 2.56	10^{-9}/L	1.80-6.30
NEUT%	0.55± 0.08	0.47± 0.12		0.40-0.75
Lymph#	4.38 ± 0.17	3.52 ± 1.36	10^{-9}/L	1.10-5.70
Lymph%	0.69 ± 0.05	0.71 ± 0.07		0.50-0.90
Mono#	0.41 ± 0.12	0.33 ± 0.05	10^{-9}/L	0.10-0.60
Mono%	0.07 ± 0.03	0.07 ± 0.07		0.03-0.10
RBC	8.14 ± 1.21	8.96 ± 0.57	10^{-12}/L	6.00-10.00
HGB	138.0 ± 5.42	117.0 ± 9.41	g/L	110-145
HCT	35.2 ± 3.73	43.7 ± 0.95		30.6-45.1
MCV	49.6 ± 4.75	49.3 ± 2.13	fL	48.2-58.3
MCH	15.4 ± 0.47	16.7 ± 2.18	pg	13.8-19
MCHC	331 ± 5.43	351 ± 2.31	g/L	326-354
RDW%	0.08 ± 0.03	0.11 ± 0.02		0.04-0.15
RDW	37.6 ± 2.18	48.1 ± 2.15	fL	37-54
PLT	336 ± 7	315 ± 16	10^{-9}/L	125-350
MPV	7.52 ± 2.36	7.71 ± 1.65	fL	4.0-12.0
PDW	12.9 ± 1.52	17.1 ± 0.15	fL	12.0-18.0
PCT	0.36 ± 0.11	0.75 ± 0.08		

Hematological parameters in whole blood of healthy SD rats following administration of probe XJYZ or PBS for 7d.(n=3)

	XJYZ	PBS	Unit	Reference Range
WBC	5.57 ± 1.21	4.65 ± 2.51	10^{-9}/L	3.50-9.50
NEUT#	5.96 ± 1.01	4.17 ± 2.36	10^{-9}/L	1.80-6.30
NEUT%	0.55± 0.21	0.41± 0.71		0.40-0.75
Lymph#	4.13 ± 0.84	3.25 ± 2.11	10^{-9}/L	1.10-5.70
Lymph%	0.35 ± 0.07	0.49 ± 0.03		0.50-0.90
Mono#	0.15 ± 0.02	0.51 ± 0.05	10^{-9}/L	0.10-0.60
Mono%	0.05 ± 0.01	0.08 ± 0.05		0.03-0.10
RBC	7.81 ± 2.13	9.73 ± 1.20	10^{-12}/L	6.00-10.00
HGB	135.4 ± 6.24	121.5 ± 4.29	g/L	110-145
HCT	36.3 ± 1.57	40.8 ± 1.75		30.6-45.1
MCV	51.3 ± 2.15	40.6 ± 5.62	fL	48.2-58.3
MCH	13.8 ± 1.25	16.5 ± 3.47	pg	13.8-19
MCHC	336 ± 5.00	341 ± 4.28	g/L	326-354
RDW%	0.04 ± 0.02	0.08 ± 0.05		0.04-0.15
RDW	39.2 ± 6.51	43.0 ± 5.21	fL	37-54
PLT	137 ± 25	255 ± 13	10^{-9}/L	125-350
MPV	6.42 ± 2.17	9.21 ± 3.80	fL	4.0-12.0
PDW	12.9 ± 1.14	17.2 ± 2.49	fL	12.0-18.0
PCT	0.57 ± 0.38	0.55 ± 0.12		

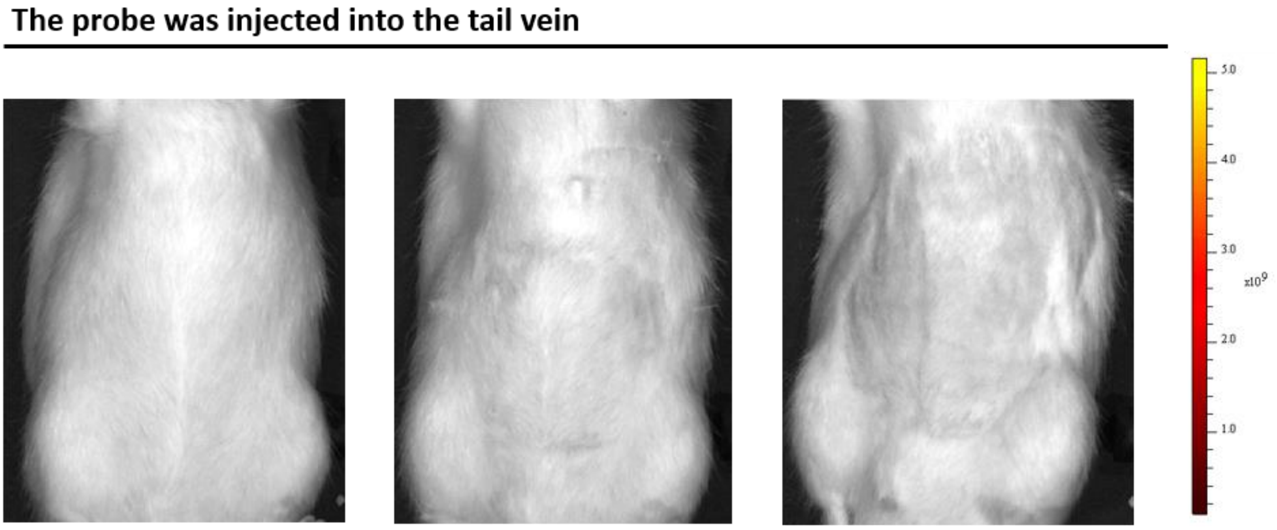

Supplementary Fig. 19. Suppression of Rat Autofluorescence

To eliminate interference from autofluorescence, in vivo fluorescence imaging was performed on recipient SD rats prior to probe injection. Organs located in the abdominal cavity showed only a slightly weak fluorescence signal, indicating that the contribution of autofluorescence to the fluorescence signal of probe XJYZ was negligible.

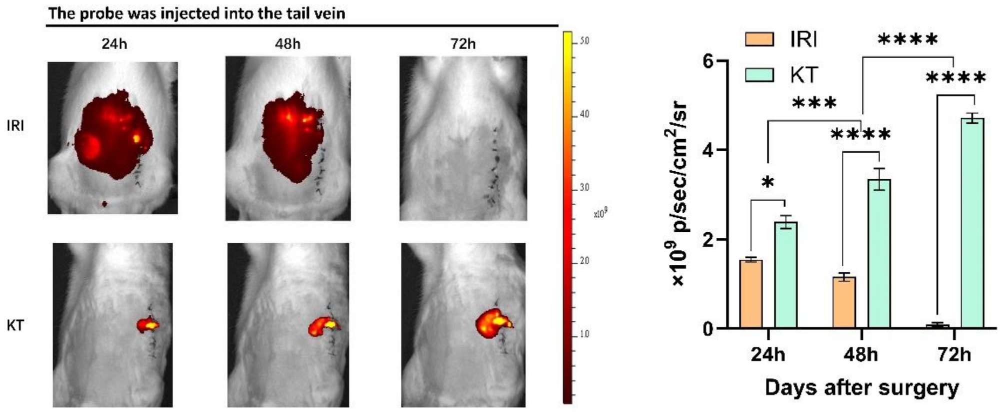

Supplementary Fig. 20. Probe imaging in ischemia-reperfusion injury

The transplantation group exhibited high-intensity fluorescence imaging within the 72-hour period; in the ischemia-reperfusion group, while imaging was observed in the left kidney at 24 hours due to the inflammatory response, the absence of subsequent rejection led to a rapid phenotypic shift in M1 macrophages and complete metabolic clearance of the probe in vivo; furthermore, owing to the inflammatory response alone, a minor portion of the probe had already been metabolized by the liver and the right kidney by 24 hours, resulting in sustained faint fluorescence intensity in these organs throughout the process; quantitative analysis revealed that the fluorescence intensity in the transplantation group was 2.23 times greater than that in the ischemia-reperfusion group.

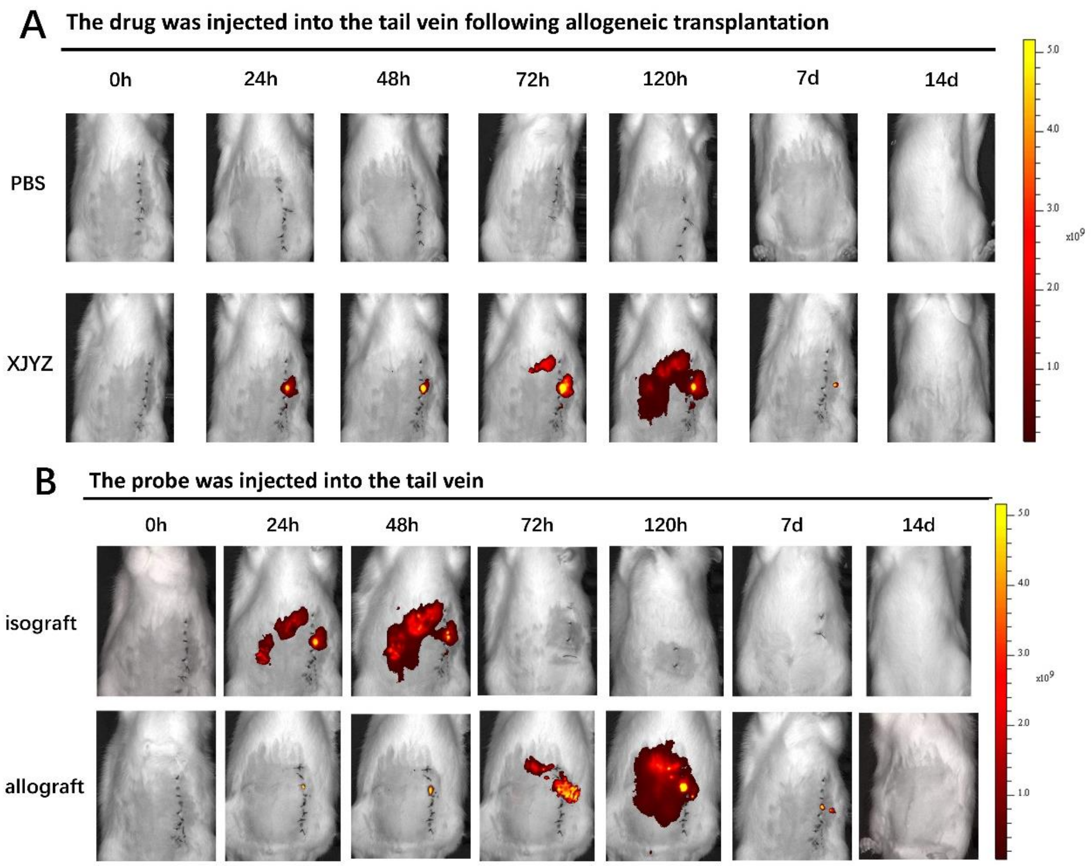

Supplementary Fig. 21. Probe XJYZ in vivo imaging

Figure A utilized allogeneic transplant recipients for both the experimental and control groups, with the experimental group receiving 1 mM XJYZ via caudal vein injection whereas the control group received an equivalent volume of PBS administered through the same route;

Figure B employed allogeneic transplant recipients for the experimental group and syngeneic transplant recipients for the control group, while both groups received identical 1 mM XJYZ injections via the caudal vein

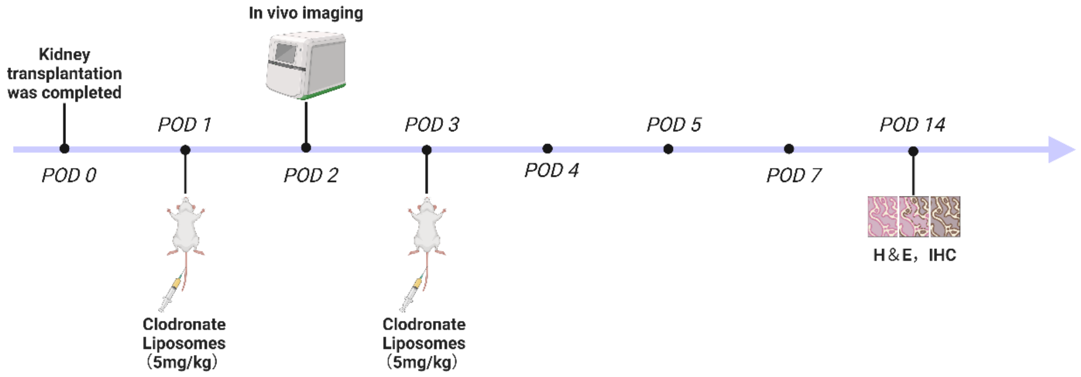

Supplementary Fig. 22. Schematic Diagram of Early Post-Transplantation Intervention

At 24h and 72h after transplantation, 5mg/kg Clodronate Liposomes was injected into the tail vein of the recipients. Imaging was performed at different time points, and the renal specimens were collected for histological study.

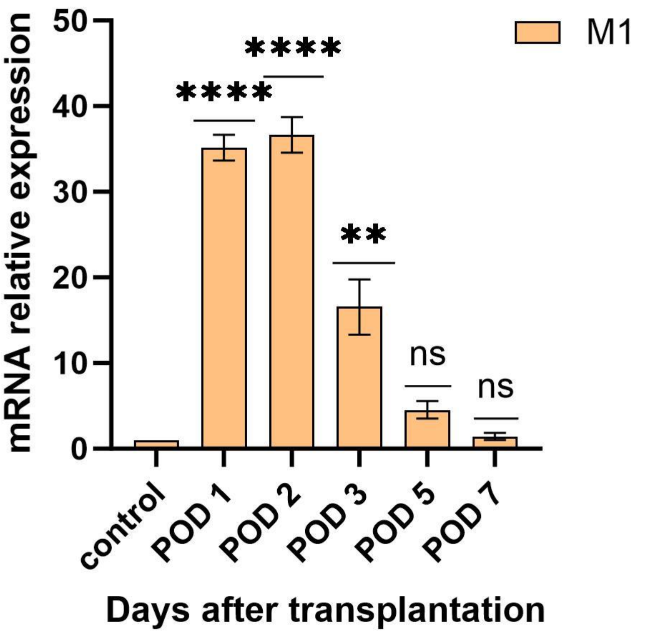

Supplementary Fig. 23. Infiltration of M1 Macrophages in Grafts After Intervention

Kidney tissues were obtained at different time points after intervention with Clodronate Liposomes, and qPCR was used to detect the expression of M1 macrophages in the transplanted kidney. Primer designs are shown in Supplementary Table 2. All bars show standard deviation ± mean (n=5).

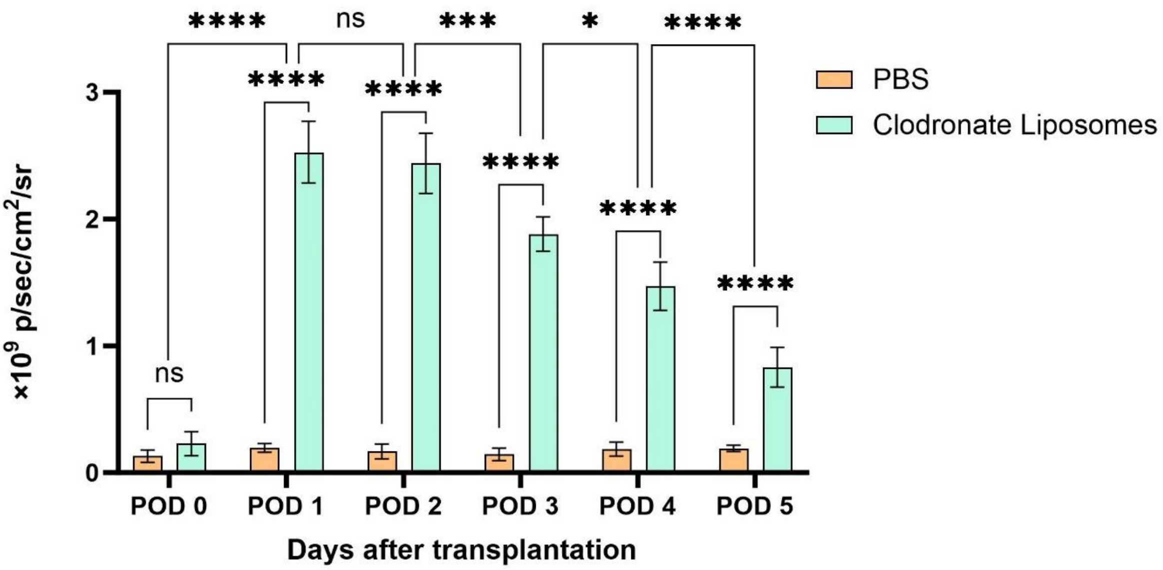

Supplementary Fig. 24. Quantification of Fluorescence Intensity in Graft Tissues After Intervention

After intervention with Clodronate Liposomes, the fluorescence quantification of the transplanted kidney site imaged by probe XJYZ in vivo at different time points was compared with the baseline fluorescence. All bars show SD ± mean (n=5).

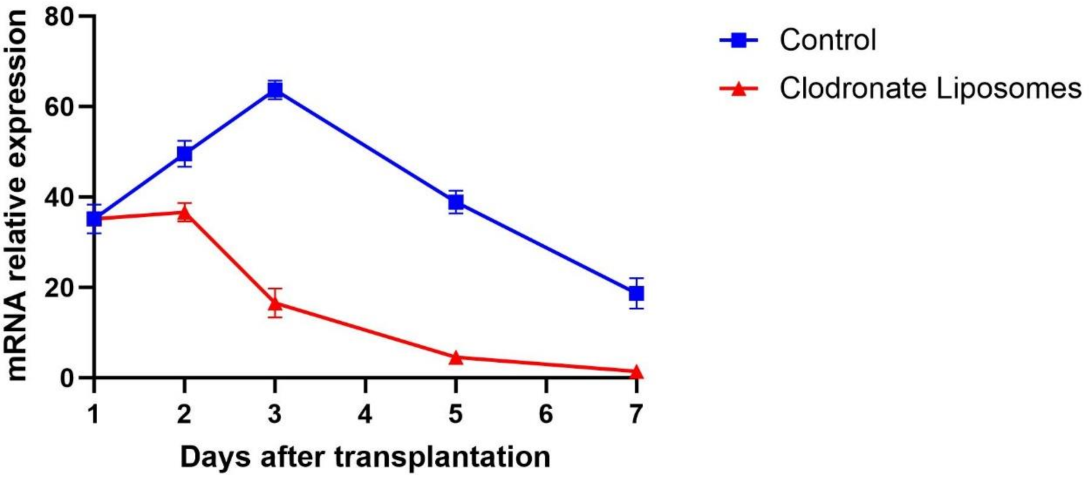

Supplementary Fig. 25. Changes in M1 Macrophage Infiltration in Renal Allografts Between Intervention and Non-Intervention Groups

The infiltration of M1 macrophages was compared between the intervention group and the non-intervention group at different time points. All data are shown SD ± mean (n=5).

Supplementary Scheme 1. Synthetic Pathway of XJYZ

XJYZ-1 (30 g, 1.0 eq.), XJYZ-2 (34.7 g, 1.5 eq.), and acetonitrile (75 mL, 2.5V) were mixed and stirred magnetically for at least 24 h. The reaction mixture was concentrated under reduced pressure, then subjected to three solvent exchanges with methyl tert-butyl ether (MTBE, 150 mL, 5V), yielding XJYZ-3 as a purple solid (36 g, purity 98.1%). Acetic anhydride (80 mL, 4V), glacial acetic acid (80 mL, 4V), XJYZ-3 (20 g, 1.0 eq.), and XJYZ-6 (20.2 g, 1.1 eq.) were combined and stirred for 2 h, followed by the dropwise addition of MTBE. The mixture was filtered and dried under vacuum to afford XJYZ-7A as a black solid (23 g, purity 70.7%). XJYZ-1 (50 g, 1.0 eq.) and XJYZ-4 (68.2 g, 1.2 eq.) were mixed and stirred for 3 h, then ethyl acetate was added. The product was isolated by filtration and drying, giving XJYZ-5 as a pink solid (36 g, purity 96.7%). Anhydrous ethanol (100 mL, 10V), XJYZ-7A (10 g, 1.0 eq.), XJYZ-5 (6.9 g, 1.0 eq.), and sodium acetate (6.6 g, 3.0 eq.) were combined and stirred for 2 h, followed by concentration under reduced pressure to obtain XJYZ-7 (7 g, purity 90.8%). Dimethyl sulfoxide (DMSO, 65 mL, 10V), XJYZ-7 (6.5 g, 1.0 eq.), XJYZ-8 (2.9 g, 1.2 eq.), PyBOP (benzotriazol-1-yloxytris(pyrrolidino)phosphonium hexafluorophosphate, 6.2 g, 1.05 eq.), and DIEA (N,N-diisopropylethylamine, 4.4 g, 3.0 eq.) were added to a reaction flask. The synthesis was conducted in an ACN/H_2O system, yielding XJYZ as a blue solid (1.3 g, purity 99.4%) after workup.

LC-MS- XJYZ : (ES, m/z): [M+H]+=658.7

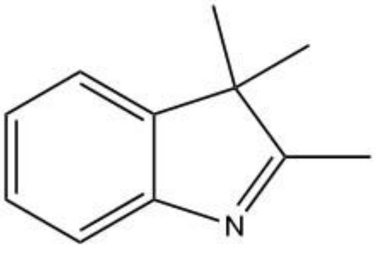

XJYZ-1

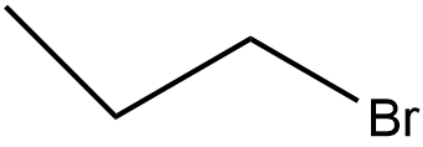

XJYZ-2

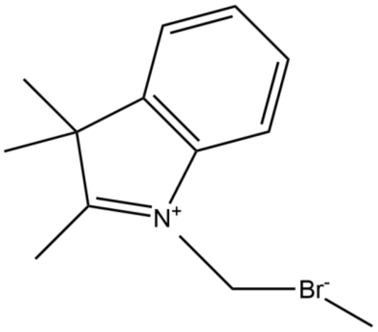

XJYZ-3

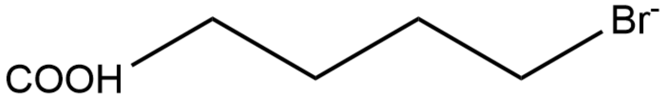

XJYZ-4

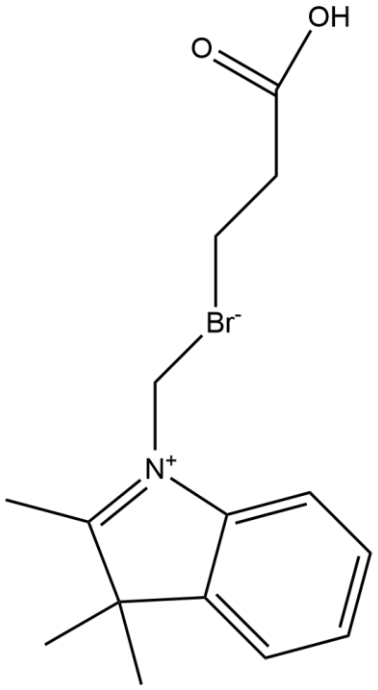

XJYZ-5

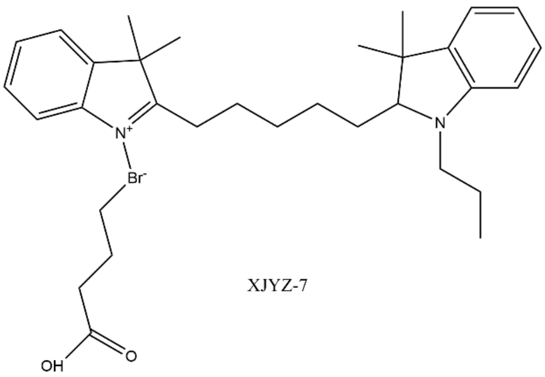

XJYZ-7

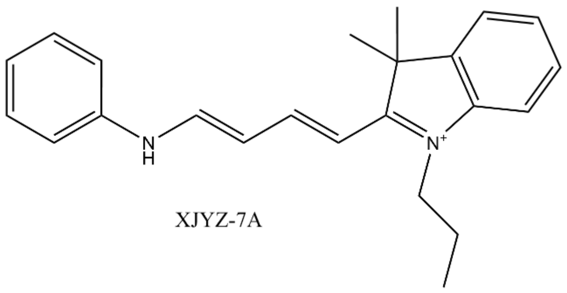

XJYZ-7A

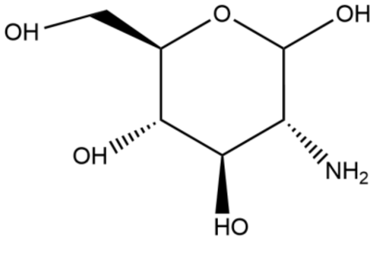

XJYZ-8

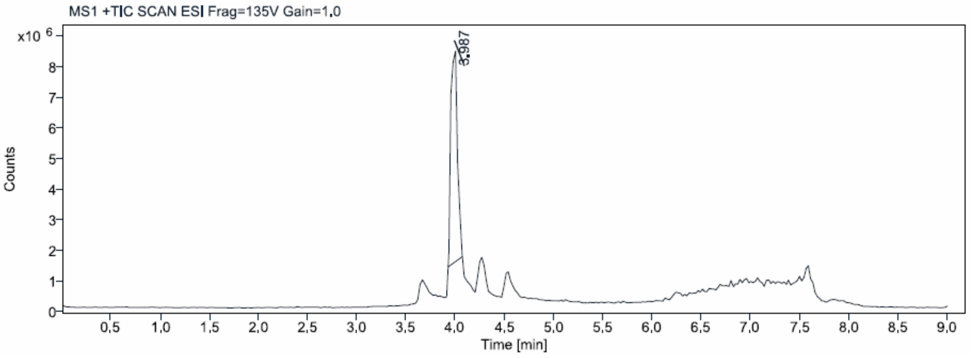

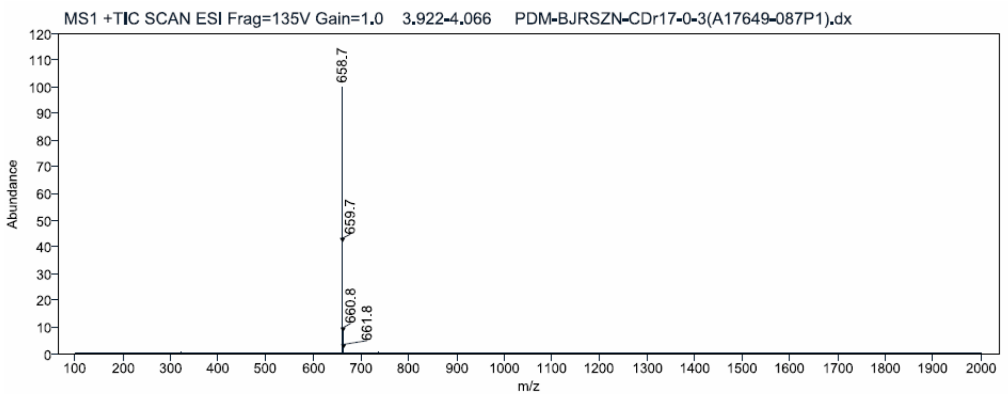

Supplementary Fig. 26.HR-MS spectrum of XJYZ.

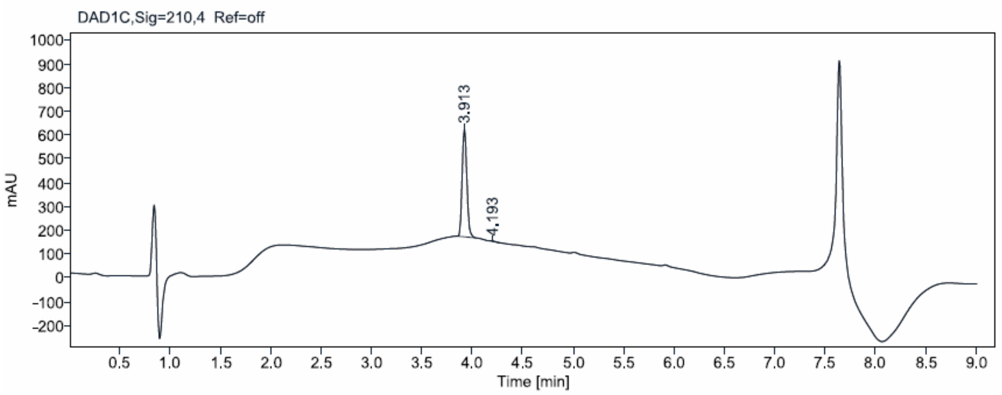

Signal: DAD1C,Sig=210,4 Ref=off

Name	RT [min]	Area	Height	Area%
	3.913	1469.0100	455.8969	99.4404
	4.193	8.2665	2.6300	0.5596
	**Sum**	**1477.2766**		

Signal: MS1 +TIC SCAN ESI Frag=135V Gain=1.0

Name	RT [min]	Area	Height	Area%
	3.987	32537922.7434	7246315.7661	100.0000
	**Sum**	**32537922.7434**		

Supplementary Fig. 27.HPLC and mass chromatogram of XJYZ.

Supplementary Table 2. Primer Sequences

Species	Gene	Fwd(5` →3`)	Rev（5` →3`）
Rat	CD86	AGACATGTGTAACCTGCACC	TTTGAGCCTTTGTGAACGGG
	GLUT1	TCAACACGGCCTTCACTG	CACGATGCTCAGATAGGACATC
	GAPDH	CACCATCTTCCAGGAGCGAG	GGCGGAGATGATGACCCTTT
Mouse	CD11b	TTGCCTCGAGGGCAGAGG	CATTCACGTCTCCCAGCACT
	CD68	GACCGCTTATAGCCCAAGGA	TCATCGTGAAGGATGGCAGG
	CD80	TTCACCTGGGAAAAACCCCC	ACAACGATGACGACGACTGT
	CD86	CAGCACGGACTTGAACAACC	CTCCACGGAAACAGCATCTGA
	CD163	GTGCTGGATCTCCTGGTTGT	CGTTAGTGACAGCAGAGGCA
	CD206	GTTCACCTGGAGTGATGGTTCTC	AGGACATGCCAGGGTCACCTTT
	GAPDH	CCTCGTCCCGTAGACAAAATG	TGAGGTCAATGAAGGGGTCGT

Supplementary Table 3. Gene set associated with M1 macrophages

cell subset	Gene set
M1 macrophages	IRF 1,CXCL 11,CXCL 9,MARCO,IL-1B,CD86,TNF-α,IL-2RA, CXCL 10,FCGR-1A

Supplementary Table 4. Western blot raw data

GLUT1	GAPDH
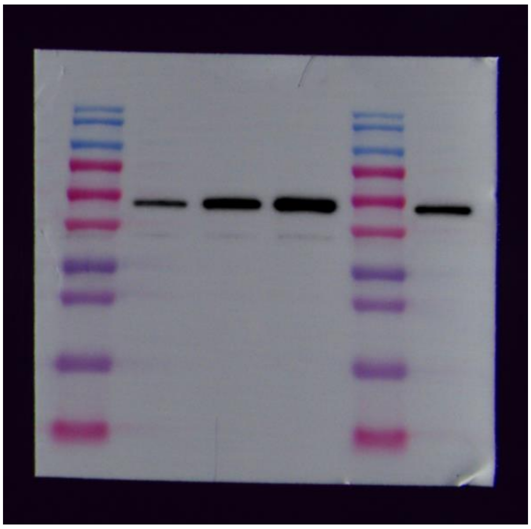	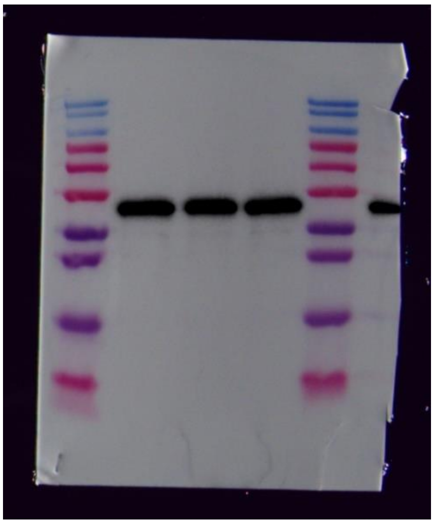

GLUT1	GAPDH
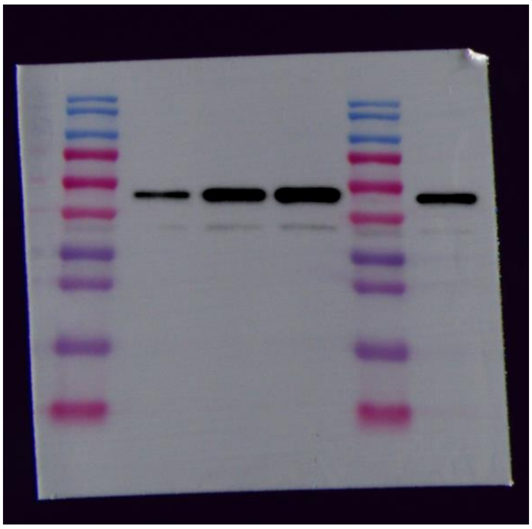	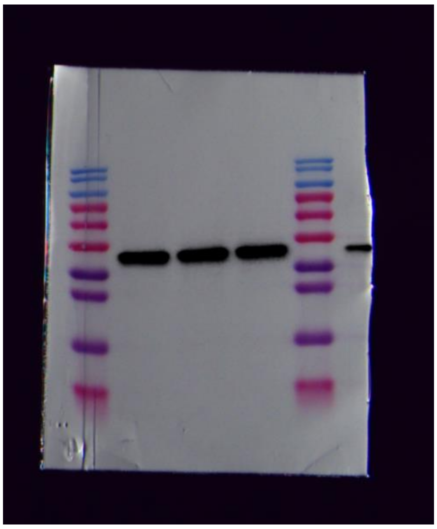
